# Synapse Geometry and Receptor Dynamics Modulate Synaptic Strength

**DOI:** 10.1371/journal.pone.0025122

**Published:** 2011-10-03

**Authors:** Dominik Freche, Ulrike Pannasch, Nathalie Rouach, David Holcman

**Affiliations:** 1 Department of Mathematics and Department of Neuroscience, Weizmann Institute of Science, Rehovot, Israel; 2 Team Neuroglial Interactions in Cerebral Physiopathology, CIRB CNRS UMR 7241-INSERM U1050 College de France, Paris, France; 3 Department of Mathematics and Computational Biology, IBENS École Normale Supérieure, Paris, France; 4 Department of Applied Mathematics, Tel Aviv University, Tel Aviv, Israel; University of Freiburg, Germany

## Abstract

Synaptic transmission relies on several processes, such as the location of a released vesicle, the number and type of receptors, trafficking between the postsynaptic density (PSD) and extrasynaptic compartment, as well as the synapse organization. To study the impact of these parameters on excitatory synaptic transmission, we present a computational model for the fast AMPA-receptor mediated synaptic current. We show that in addition to the vesicular release probability, due to variations in their release locations and the AMPAR distribution, the postsynaptic current amplitude has a large variance, making a synapse an intrinsic unreliable device. We use our model to examine our experimental data recorded from CA1 mice hippocampal slices to study the differences between mEPSC and evoked EPSC variance. The synaptic current but not the coefficient of variation is maximal when the active zone where vesicles are released is apposed to the PSD. Moreover, we find that for certain type of synapses, receptor trafficking can affect the magnitude of synaptic depression. Finally, we demonstrate that perisynaptic microdomains located outside the PSD impacts synaptic transmission by regulating the number of desensitized receptors and their trafficking to the PSD. We conclude that geometrical modifications, reorganization of the PSD or perisynaptic microdomains modulate synaptic strength, as the mechanisms underlying long-term plasticity.

## Introduction

Synapses are local micro-contacts between neurons mediating direct neuronal communication via neurotransmitters. Several well-identified processes are involved in synaptic transmission, such as the release of neurotransmitters from the presynaptic terminal into the synaptic cleft. This vesicular release results in the activation of receptors located on the postsynaptic neuron. At excitatory synapses, open receptors such as AMPARs, a class of glutamate gated channels, mediate neuronal depolarization by an ionic current. The postsynaptic response depends on several factors [1]–[3] such as the number of release synaptic vesicles, the release probability at the presynaptic terminal, the synaptic cleft geometry, the glial coverage and the number and distribution of postsynaptic receptors that determine the time course of neurotransmitter activity. Thus, if synaptic transmission at a single synapse over time depends on so many stochastic events, how can the synaptic signal be reliable?

Previous computational studies of synapses with stationary receptors [3]–[9] show that several geometrical features such as cleft height and localization of vesicular release contribute to shaping the postsynaptic current over time. So far, only a few quantitative results are known about the characteristics of receptor trafficking, which may affect synaptic transmission [10]–[14]. Furthermore, it is unclear whether fluctuations in PSD receptor density affect the amplitude of the synaptic current at a time scale that could interfere with fast spiking. Indeed, recent findings indicate that receptor trafficking has a fast functional implication on synaptic transmission [10]–[14]. If the number of receptors can vary at the PSD, moving with a diffusion constant in a range of 0.1 to 0.2 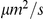 [13], then this motion may affect the amplitude of the synaptic current and fast spiking of about 20 Hz. Because extrasynaptic receptors could potentially replace synaptic ones, in particular those desensitized by glutamate molecules, a refined combination of experiments led to the proposition that receptor trafficking has a fast functional implication on synaptic transmission [10]–[16]. This was illustrated in a paired-pulse protocol where, in the absence of receptor diffusion, the second pulse was diminished [17].

To investigate how vesicles and receptor location, cleft geometry, receptor trafficking, and recycling as well as glial coverage influence the temporal expression of the postsynaptic current, we develop here a computational model to simulate the different steps of synaptic transmission, starting from vesicle release. To account for the Brownian motion of receptors, neurotransmitters dynamics and receptor opening and closing, we use Markov chain modeling and present results from Brownian dynamics simulations. However, we do not construct here any fitting procedure. Our approach allows simulating synaptic transmission based on the molecular properties of receptors and the geometrical organization. We built a synapse with a cleft surrounded by astroglia which take up glutamate molecules through transporters. On the postsynaptic terminal, receptors can move by lateral diffusion and enter the PSD, where they can be trapped by scaffolding molecules. In our model, PSD receptors are maintained at equilibrium with a pool of extrasynaptic receptors inside a reservoir, isolated from the rest of the dendrite. We refer to perisynatic and extrasynaptic areas, as the microdomains surrounding the PSD, and outside the PSD, respectively.

We first quantify the role of synapse geometry on synaptic transmission and then show that although receptor desensitization contributes to paired-pulse depression, receptor diffusion can restore the second pulse by about 5% at 25 Hz, and by 20% with further stimulations (at least 10 pulses). Second, to determine the conditions for which the synaptic current is maximal, we analyze the relative position of the PSD versus the active zone (AZ) where vesicles are released. We find that an alignment of vesicle release sites and a high concentration of receptors on the PSD, which is possibly mediated by adhesion molecules [18], leads to a maximal current. Finally, we study the consequence of spike correlation on synaptic transmission. We show that a low vesicular release probability can decorrelate spikes (for a frequency larger than 10 Hz). Moreover, increasing the inter-spike interval has several consequences: We find that when a vesicle is successfully released at a single synapse, it depresses the AMPARs. Thus, by reducing the release probability (by five), many spikes will not be generated which prevents AMPARs from becoming desensitized. As a consequence of this filtering, we show that a successfully released vesicle on average leads to a fivefold higher current compared to a situation where the release probability is one (no filtering). However, the price to pay is to filter spikes (take one in four) at the synaptic level. We show that it is actually an advantage that synapses are unreliable in order to produce a detectable and significant synaptic current. Neurons can overcome this local inherent unreliability by making multiple synaptic boutons [19] to the targeted neuron.

## Results

**Figure 1:**
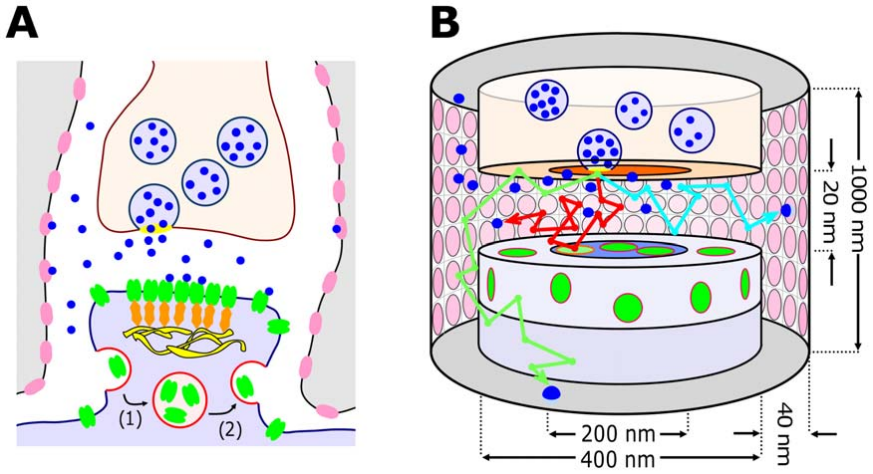
Representation of the synapse dynamics. (A) Sketch of an excitatory synapse consisting of the presynaptic terminal where vesicles are released, and the postsynaptic element where glutamate receptors are located. The synapse is surrounded by astroglial processes containing glutamate transporters (GLTs). Presynaptic vesicle fusion occurs at randomly selected locations, released glutamate (blue) diffuses in the cleft and binds to AMPARs (green) or GLTs (pink). AMPARs diffuse between the PSD, where they can attach to scaffolding molecules (orange) and the extrasynaptic regions, where they can undergo endocytosis (1) and exocytosis (2), maintaining the number of AMPARs at the post-synaptic terminal. (B) Two co-axial cylinders represent the pre- and postsynaptic terminal, forming a gap which represents the synaptic cleft. AMPARs (green) are distributed inside and outside the PSD. The trajectory of a glutamate molecule as illustrated by red, blue or green arrows corresponds to binding to AMPARs, GLTs or diffusing away from the cleft (at 500 nm), respectively.

We approximate the synaptic cleft as two coaxial cylinders (see Fig. 1) where AMPARs are distributed on the PSD and in a perisynaptic microdomain modeled as a reservoir surrounding the PSD. Receptors can move by free diffusion and can be exchanged between these two regions. Glutamate molecules are released after vesicle fusion, which may occur at release sites placed anywhere on the presynaptic terminal. Finally, transporters are distributed uniformly on the glial sheath surrounding the synapse.

### Effects of synaptic geometry, vesicular release location and glial transporters on open AMPARs

**Figure 2:**
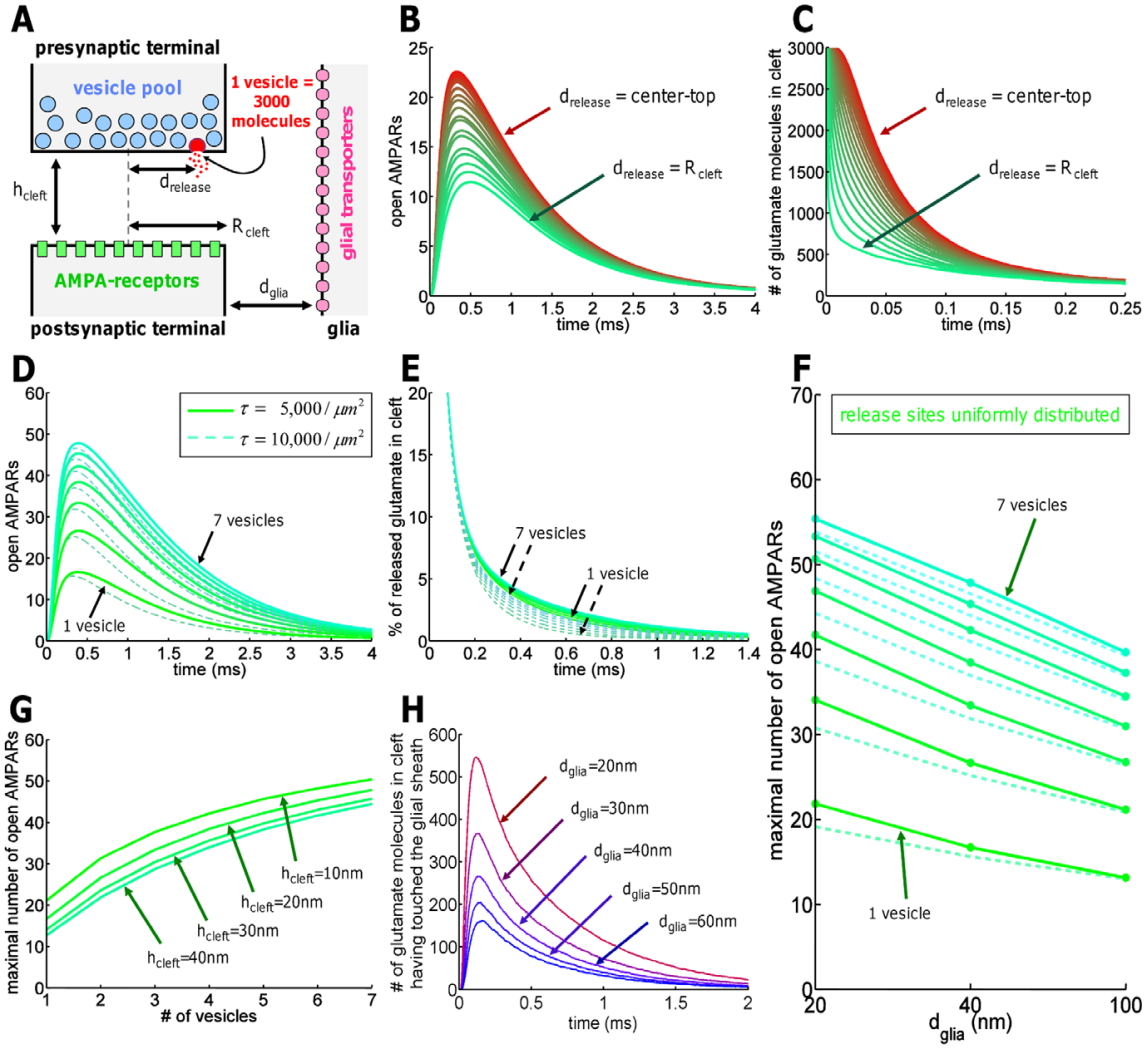
Dynamics of the cleft. (A) Schematic representation of the synapse: cleft height 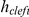 = 20 nm, 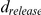 denotes the distance of vesicle release from cleft center, 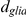 = 40 nm is the distance from the glial sheath to the cleft exit, glial transporter density 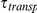 = 5,000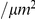, cleft radius 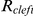 = 200 nm, 130 AMPARs are uniformly distributed on the postsynaptic terminal. The vesicle release sites were uniformly distributed on presynaptic terminal inside the cleft. (B, C) Impact of variation of release site relative to the receptor location on the number of open AMPARs (B) and glutamate concentration in the cleft (C): Release site distance was varied in steps of 10 nm from the edge (200 nm, green) to the center (0 nm, red) of the AZ. (D, E) Doubling 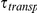 from 5,000 to 10,000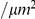 has little influence on peak open AMPAR numbers (D) and on glutamate molecules (E), but accelerates the time course of receptor closing. (F) Changing 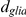 = 20 nm, 40 nm, 100 nm affects the maximal number of open AMPARs (simultaneously released vesicles: from 1 to 7, 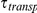 is 5,000 (solid) or 10,000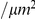 (dashed)). Transporters maximally influence transmission for small 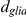 and low number of released vesicles. (G) Increasing 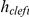 from 10 nm to 40 nm decreases the number of open AMPARs. (H) Influence of glial cells on synaptic transmission: Glutamate molecules re-entering the cleft after hitting the glial cell (no transporters, 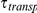 = 0), for 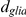 = 20 nm to 60 nm.

Although the role of several geometrical parameters have already been explored on AMPAR-mediated synaptic current [20]–[22], we present here an integrated and unified model, that first confirmed previous results, validating our approach, and then provide new quantifications and predictions. To study the impact of geometrical parameters on synaptic transmission, we follow the dynamics of open AMPARs. To quantify the effect of vesicular release, we release them at increasing distances (in steps of 10 nm) from the center of presynaptic terminal. 130 receptors are uniformly distributed over the postsynaptic neuron. Astrocytic processes are located at a distance of 40 nm away from the synaptic cleft edge (Fig. 1) and contain a transporter density of 5,000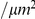 [23]. The cleft height is 20 nm. Classically, AMPAR can be in one of three states, which can be further subdivided by sub-conductance states, accounted in Markov models [21], [24]: A receptor can either be open (a current can flow), closed, or desensitized (the receptor is closed and does not respond to any glutamate stimulation). One intermediate state is for example called deactivation, the closing of the receptor and subsequent unbinding of the ligand, as opposed to receptor desensitization (i.e., the ligand remains bound to the receptor in a long-lasting nonconducting state). The transitions between sub-conductance states have been described by Markov models (see Text S1). To evaluate the number of open AMPARs, we use two well-known AMPAR models: the Milstein-Nicoll (MN) and the Jonas-Sakmann (JS) schemes [24], [25] (see Figure 2 in Text S1). We also tested another scheme, presented by Raghavachari-Lisman [25] (RL scheme) (Fig. 6). Here, all presented numbers are obtained with the MN scheme unless marked otherwise. These schemes differ by their number of states and rate constants. Although an AMPAR has four potential binding sites, the JS accounts for only two, while MN only for one. The RL scheme accounts for the four subunits, but not for the different AMPAR subunits accounted for by the the MN scheme, which was obtained by fitting recent data from the GluR4 AMPAR subunit without TARP ligation [25]. One of the main striking differences between the MN and JS schemes is the average time an AMPAR spends in the desensitized states (see Text S1 for a detailed quantification). Using these schemes, we study the number of open AMPARs and show that it decreases drastically as a function of the release site distance (Fig. 2B): the minimum and maximum numbers of open AMPARs are approximately 11 and 23 out of 130 respectively (MN) (JS scheme: 7 resp. 22). (Fig. 2A). Correspondingly, the synaptic glutamate concentration rise disappears in less than 0.3 ms (Fig. 2C). For a release distance 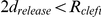 (the radius of the postsynaptic terminal, measured from the center), the decrease in open AMPARs is less than 30%, whereas for 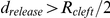, the change is drastic (divided by 2). Because the location of vesicular release matters, we systematically test two types of release site distribution: one with all sites placed in the center and another one with all sites uniformly distributed.

We next study how the number of open AMPARs depends on the number of vesicles released (Fig. 2D) and on glial glutamate transporters. To explore various activity regimes, we release up to seven vesicles at uniformly distributed release sites for two different transporter concentrations (5,000 and 10,000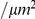). After seven vesicles, the number of open receptors saturates at about 40% for the MN scheme (JS scheme: 50%), as reported [22]. However, doubling the transporter density on glia does not affect the maximal number of open AMPARs, as previously found [22]. To confirm that the direct effect of transporters can be neglected when the vesicles are released at the center, we estimate the number of glutamate molecules returning into the synaptic cleft after escaping (Fig. 2H): at 40 nm, the number is around 250, which is less than 10% of the free glutamate molecules. Moreover, the relative clearance of glutamate molecules for one or seven vesicles is of the same order (Fig. 2E). We summarize in Figure 2G the number of open receptors as a function of the number of released vesicles for different cleft heights, known to change during development and pathological condition [26], [27].

Finally, we estimate how the synapse-to-glia distance affects the number of open AMPARs and ran simulations for glial distances of 20 nm, 40 nm and 100 nm, where one to seven vesicles are released uniformly distributed over the cleft, and for two transporter densities 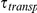 of 5,000 and 10,000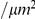. In Figure 2 of Text S1, we present the results for release sites centered on the AZ. For a transporter density of 5,000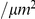, changing the glial distance from 20 nm to 100 nm of the edge of the synapse to the glial sheath (a range measured in [28]) reduces the mean maximal number of open AMPARs by 27% (JS: 33%) for a single vesicle released in the center, while reduction reaches 40% (JS: 46%) for uniformly distributed release sites over the presynaptic terminal. For seven released vesicles, the reduction becomes 28% (JS: 22% and 37%). For a glial distance of 20 nm, doubling the density of transporters reduces the number of open AMPARs by 13% (JS: 16%). For seven vesicles, reduction is 3% (JS: 4%). However, no reduction effect is found for a glial distance of 100 nm. We conclude that in all cases, changing the glial distances in a range of 20 to 40 nm will maximally affect the number of open AMPARs by 24% (JS scheme: 28%). When the release sites are located in the AZ, this number is changed by 15% (JS scheme: 20%, see Text S1).

### Glutamate transporters limit glutamate spread up to 500 nm from the synapse

**Figure 3:**
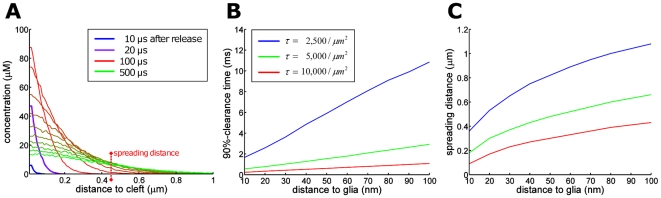
Glutamate dynamics in the extracellular space. (A) Plot of the glutamate density in the extrasynaptic space for various times after vesicular release. Glial distance is 40 nm and transporter density is 5,000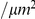. (B, C) Clearance time and spreading distance is shown for various glial sheath distances from 10 nm to 100 nm and transporter densities from 2,500 to 5,000 to 10,000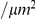.

Efficient removal of glutamate from the extrasynaptic space is crucial to limit spillover and desensitization of synaptic AMPARs. To analyze the extent of glutamate spread in the extrasynaptic space, we simulate freely diffusing glutamate molecules between two concentric cylinders for various glial transporter densities. For a synapse-to-glia distance of 40 nm and a transporter density of 5,000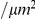, 90% of the released glutamate is bound in one ms within a distance of 0.42 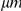 away from the releasing synapse (Fig. 3A), confirming that spillover does not activate neighboring synapses [5]. To study the influence of transporter density, we estimate the time in which 90% of the released glutamate is taken up by transporters (clearance time) and the maximal distance beyond which the glutamate concentration is 10% of the amount released (spreading distance). We find (Figs. 3B,C) that the clearance time remains on the order of a few milliseconds and the spreading distance can reach the mean distance between two neighboring synapses of around 0.5 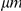 [29] (Results for a doubled glutamate diffusion constant are shown in Figure 3 in Text S1).

### Optimal synaptic transmission for alignment of PSD and active zone

**Figure 4:**
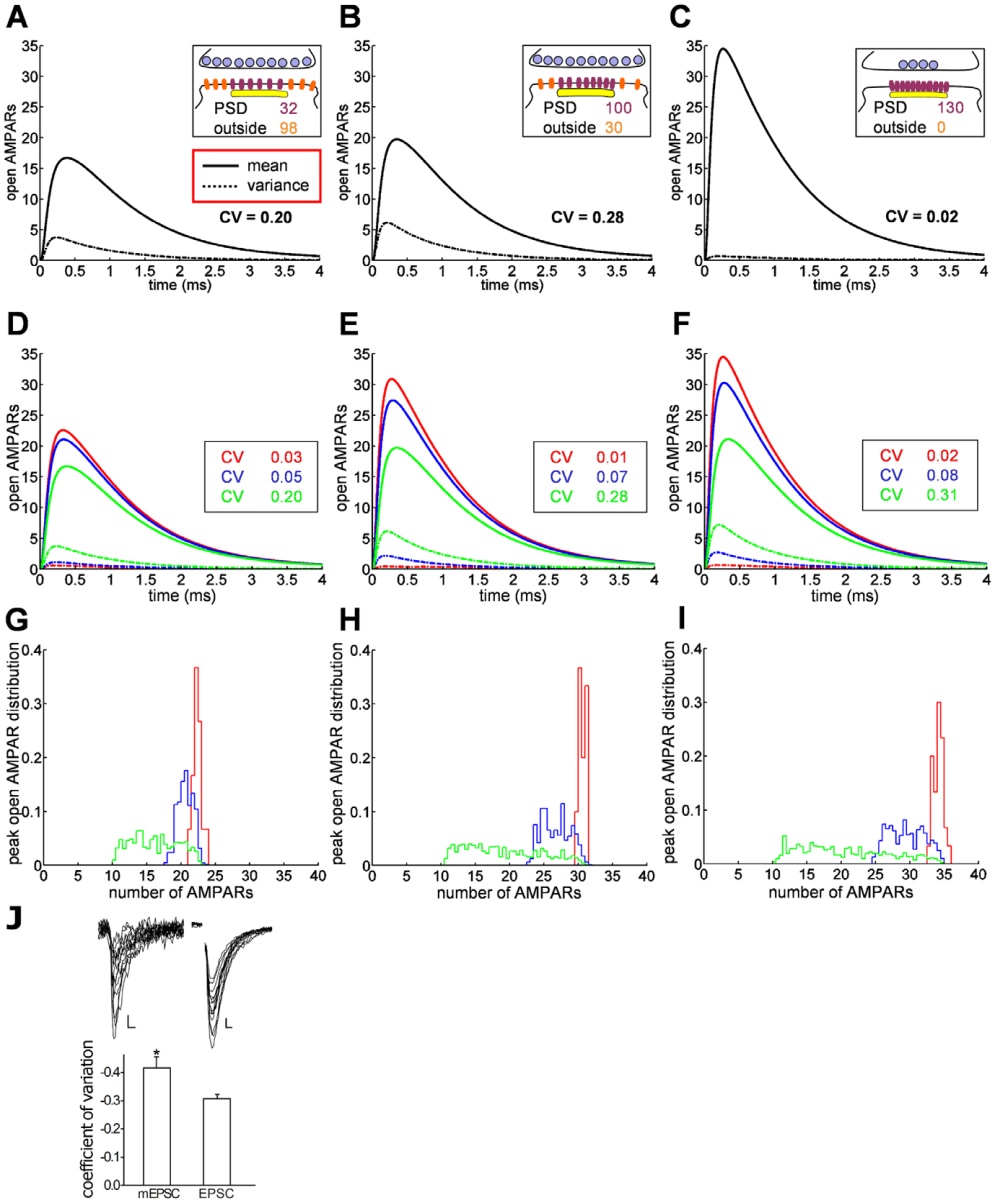
Increased efficiency of synaptic transmission by alignment of release sites and receptors. Figures (A) to (F) show the mean (solid line) and variance (dashed) of the number of open AMPARs for different configurations of vesicle release sites and AMPARs: (A) for vesicle release sites and AMPARs uniformly distributed (UD) over AZ and PSD, respectively, (B) for UD release sites but AMPARs clustered at the PSD, (C) for release sites and AMPARs clustered at the AZ center and the PSD, respectively. In that case, the CV is divided by 10, while the mean number of peak open AMPARs increases from 15 to 20 to 35. (D–F) The number of AMPARs for different release distributions (red: release in the center of the AZ; blue: release sites UD over PSD; green: release sites UD over the presynaptic terminal). (G–I) The distributions of the number of peak open AMPARs, corresponding to the different release site and receptor localizations. (J) The coefficient of variation of AMPAR-mediated peak amplitudes of miniature EPSCs (n = 8) is larger compared to evoked EPSCs (n = 15, P
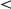
0.01). Representative sample traces of AMPAR-EPSCs (Scale bar, 10 pA, 5 ms) and mEPSCs (Scale bar, 5 pA, 5 ms) are shown above the respective bars.

The structural organization of the synapse is fundamental for synaptic transmission, and to analyze the functional consequence of the localization of the PSD relative to the active zone, we estimate the number of AMPARs activated in three cases: 1) when both vesicle release sites and AMPARs are uniformly distributed (UD) over the pre- and postsynaptic terminals, 2) for UD release sites but AMPARs concentrated on the PSD, and 3) for both release sites and AMPARs concentrated at the AZ and PSD, respectively. In the last case, AMPARs and release sites are exactly centered and apposed. In Figures 4 A,B,C, we show the mean and the variance of the number of open AMPARs.

As receptors and release sites become co-localized, the coefficient of variation (CV) decreases by the factor 10, while the mean number of open AMPARs increases from 17 to 35. We also show that the number of open AMPARs depends on release site distributions and is higher when release sites face the postsynaptic AMPARs (Figs. 4 D to 4 I). Interestingly, UD release sites may represent miniature EPSCs, described as spontaneous vesicular release events [2], whereas release triggered by an action potential may cause vesicle fusion in the AZ apposed to the PSD. Indeed, our whole cell recordings of hippocampal CA1 pyramidal cells reveal a higher coefficient of variation for mEPSCs than for evoked EPSCs (Fig. 4 J). Figures 4 D,E,F show that UD vesicular release is responsible for a much smaller number of activated AMPARs but with larger variance. The CV between UD and AZ-centered release differs by a factor 10. By definition, the CV computed here does not account for any variability in vesicle release probability. Comparison of this simulated CV with experimental data requires to only take successful synaptic events into account for the data set. In this way, any changes occurring in the CV can be related to a variation in vesicle release position or in post-synaptic dynamics. We conclude that this source of fluctuation is due to the randomness of vesicle release location relative to the PSD and very little due to receptor trafficking. Moreover, receptor clustering leads to a more reliable transmission (CV is minimal), suggesting that PSD placement plays a fundamental role for the synaptic current.

### Synaptic efficiency by AMPAR relocation from extrasynaptic to synaptic sites

**Figure 5:**
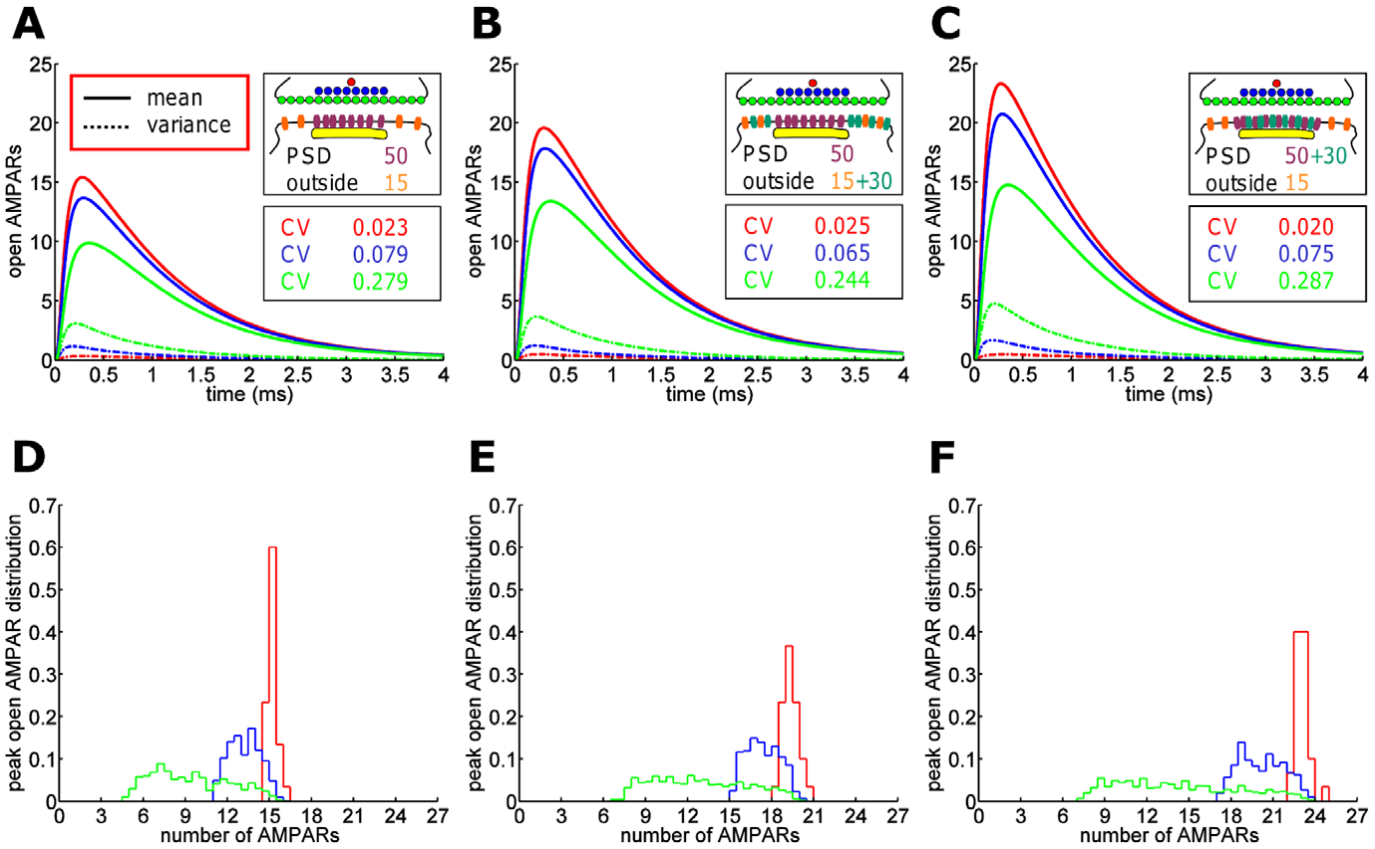
Increase in AMPAR density following long term potentiation. (A–C) An equilibrated synapse (A) transits, by extrasynaptic AMPAR insertion (B), to a synapse with an increased number of PSD-based AMPARs (C). Insertion of receptors leads to a 27% increase in the number of open AMPARs (B). Translocation of these receptors to the PSD results in a further 23% increase (C). This transition can be viewed as a two-step process following LTP where at the beginning receptors are apposed to the presynaptic area but are not inside the PSD. (D–F) The distribution of the synaptic response corresponding to the synaptic settings of (A), (B), (C), where (D) corresponds to (A). Three different release site distributions were simulated: release at the the AZ center (red); release sites uniformly distributed over the AZ (blue); release sites uniformly distributed over the entire presynaptic terminal (green). The current variation is more reduced for release at the AZ center or for a small active zone compared to a uniform release. (Glial transporter density: 5,000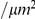.).

Synaptic plasticity at CA1 Schaffer collateral synapses has been attributed to the local change in the number of AMPARs, because long-term potentiation increases the AMPAR density [11]. This increase occurs at the PSD and may also concern the extrasynaptic space. To study the consequence of AMPAR spatial organization on the synaptic current, we increase AMPAR number by 50% by inserting additional AMPARs inside or outside the PSD (Figs. 5 A,B,C). The first case leads to a 27% increase in the number of open AMPARs (from 15.4 to 19.6) for an AZ covering the PSD, while increasing AMPARs directly at the PSD leads to a 50% increase (from 15.4 to 23.3), confirming the critical role of the density of AMPARs for synaptic transmission [11], [22]. We further investigated the consequence of different vesicle release site locations: at the center of AZ, uniformly distributed (UD) over the AZ and UD over the presynaptic terminal. Figures 5D,E,F show the corresponding dispersion for the three release site distributions. The spread distribution corresponding to vesicle release over the presynaptic terminal is one of the main sources of synaptic current fluctuation. We conclude that adding AMPARs is the most efficient way to increase the synaptic current as demonstrated experimentally in [15], [16], and translocation of receptors from the extrasynaptic pool to the PSD leads to a 23% increase, while the CV remains approximately constant, showing that the mean and the standard deviation vary equally with changes in receptor number. Thus, we predict that there will be no alteration of the synaptic current variation (CV) during synaptic plasticity, if these changes occur only postsynaptically. Therefore, we attribute changes in CV of evoked EPSCs, which were experimentally measured before and after LTP, to modifications other than those considered here, such as changes in release probability. We conclude from this analysis, that LTP may be viewed as a two-step process, in which at first receptors are inserted extrasynaptically and then traffic to the PSD to attach to scaffolding molecules, with an increase of 27% in the first step and an additional 23% in the second, leading to an approximate total increase of 50%.

### Receptor trafficking significantly modulates synaptic transmission only after a pulse train

Because receptors can move in and out of the PSD [12]–[14], we look at the effect of receptor trafficking on synaptic transmission. After two and more consecutive pulses, we estimate the number of open AMPARs. After a single vesicle release, receptors can be either closed, open or desensitized. In the latter case, the amplitude of the synaptic response elicited by a second pulse will be reduced unless they are replaced by non-desensitized receptors entering from outside the cleft by diffusion.

**Figure 6:**
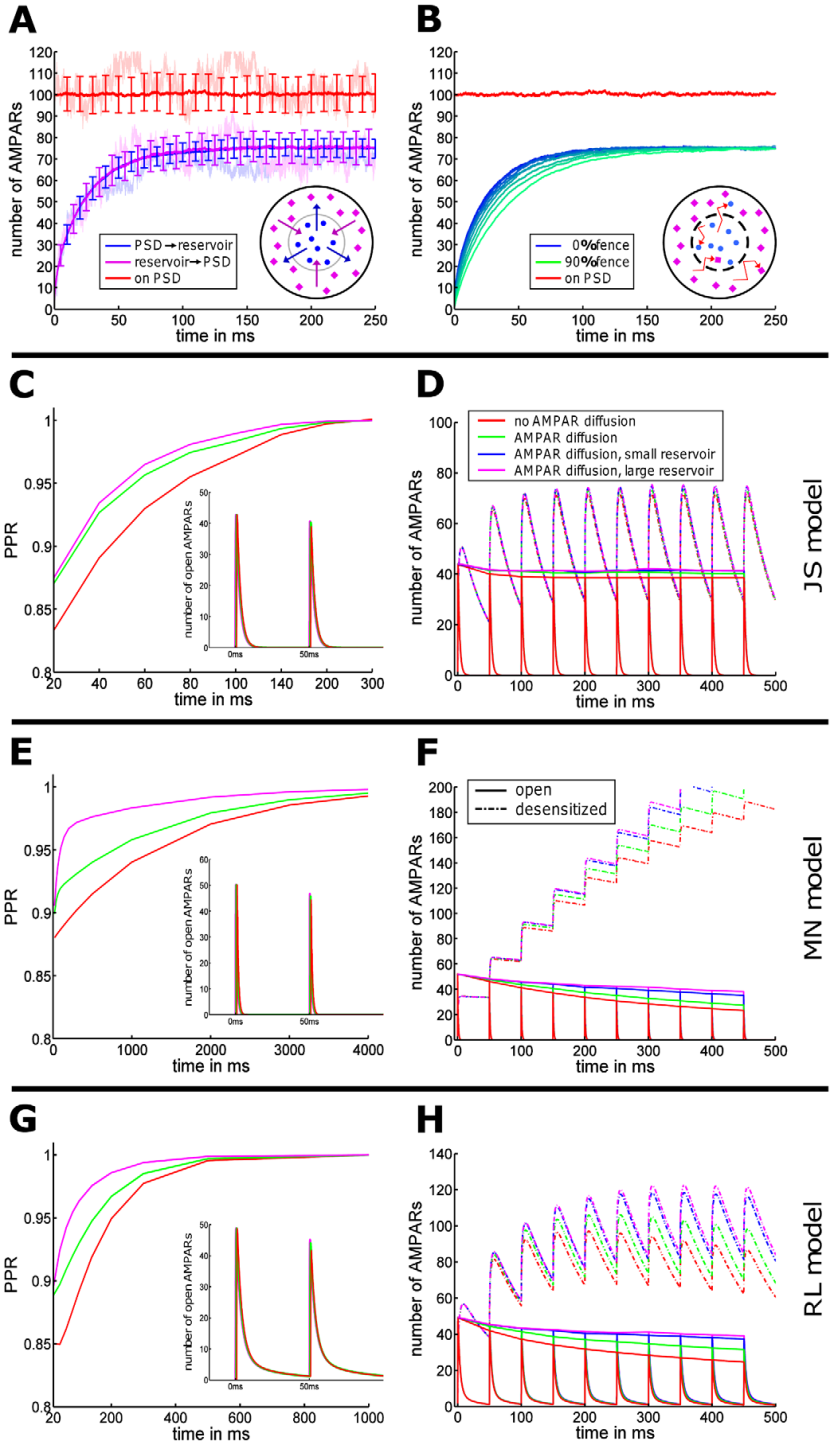
Effect of receptor trafficking on synaptic transmission. (A) For a PSD (diameter 200 nm with 100 AMPARs marked blue at time 0) and an outside reservoir (diameter 400 nm with 300 AMPARs marked pink at time 0), the time course of AMPAR exchange by receptor diffusion (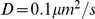) is shown. Within 50 ms the two AMPAR populations (blue and pink lines) are equilibrate to 75%, while the average number of AMPARs on the PSD remains constant. Error bars: variance, light colors: sample trajectories. (B) The time course of exchange is shown for a PSD with partially impenetrable boundary. Despite placing 10 equally-spaced barriers (indicated in the inset by the dashed circle) covering 0 (blue) to 90% (green) of the total PSD boundary, the mean number of receptors (red) inside the PSD does not change. (C, E, G) Stimulation with two consecutive pulses (frequency ranging from 20 Hz–0.4 Hz), each leading to the release of 1 vesicle in the center of the AZ in either presence or absence of AMPAR diffusion. The paired-pulse ratio (PPR) is shown at the maximal number of open AMPARs for the JS (C), MN (E) and RL model (G). The effect of AMPAR diffusion on the PPR was maximal for 20 ms. (D, F, H) During 10 pulses of a 20 Hz pulse train, the number of diffusing (green) and immobile (red) open AMPARs decays, while the number of desensitized AMPARs increases (dashed).

At steady state, receptors are exchanged between PSD and reservoir and we design a synapse with equal receptor density in PSD and reservoir such that, on average, receptors are maintained at a number of 100 on the PSD and 300 in the reservoir. In that case, the mean and variance of the receptor number on the PSD are
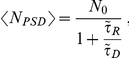


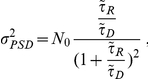
where 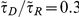 is the steady state ratio of the PSD and reservoir resident times such that 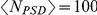 and 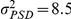. In Figure 6 A, we show a realization of the receptor dynamics inside the PSD. Mean and variance are obtained by averaging over 50 realizations. Because an aggregation of impenetrable obstacles constitutes a corral area which restricts the motion of receptors and confines them, we decided to implement a fence (a wall with some small holes) around the PSD. A receptor is then reflected by the fence and thus can stay a longer time in the PSD (see Section 7.2.3 in Text S1 for the implementation). We first performed a simulation for unrestricted diffusion at the PSD (no fence). In this case, the resident time of a receptor in the reservoir (resp. PSD) is 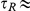
48 ms (resp. 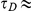
12 ms) (averaged over 100 runs). Then, to study receptor exchange between PSD and reservoir, we plotted the time course of receptors arriving at and leaving from the reservoir. The mean number of exchanged receptor is given by (see Section 1 in Text S1)
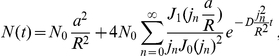
where 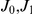 are the Bessel functions of the first kind of order zero and one, respectively, and 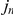 are the ascending zeros of 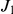. For a small PSD, and for a time 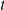 larger than a few milliseconds,
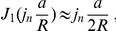


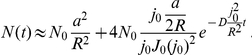
In our simulations, the boundary of the reservoir is impenetrable for dendritic receptors. For a PSD diameter of 200 nm, cleft and reservoir diameter of 400 nm, we find that receptors from the reservoir can replace, within 50 ms, 80% of the PSD free receptors. The increased variance of the receptor number entering the PSD compared to the one entering the reservoir is due to the difference of the reservoir size compared to the PSD area (factor 3). After a sufficiently long time (100 ms), the receptor number at the PSD is lower than the number at equilibrium, because a fraction of these receptors remains in the PSD. These recovery curves simulate FRAP experiments, where bleached receptors leave the PSD and are replaced by extrasynaptic ones. In Figure 6 B, we show the time course of replenishment for different fractions of PSD fence (from 0 to 90%). The fence slows down the receptor exchange, but after 50 ms, a fence coverage of 0% compared to 90% does affect the speed of receptor replenishment. We conclude that only large fence coverage of more than 90% can change the transient time course. At a 90% fence coverage, the resident time in the PSD (resp. reservoir) of a receptor is 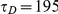 ms (resp. 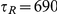 ms), in agreement with the resident time formula [30]. We note that if 90% fence coverage is made of 10 fence parts, 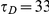 ms and 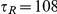 ms. We conclude that a receptor cannot be confined inside the PSD for time of the order of minutes just by a fence unless it is bound to scaffolding molecules [31].

To test the functional role of diffusion on synaptic transmission, we use a paired-pulse protocol (Fig. 6 C,E,G) in which two vesicles are released successively in the synaptic cleft at the center of the presynaptic terminal with a time delay of 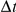 = 50 ms. This protocol does not account for any facilitation mechanism. When no corral is present, we either allow receptors to diffuse (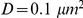/s) or not. We use the JS and MN schemes for AMPAR dynamics: In all cases, receptor diffusion increases the amplitude of the second pulse by about 10%. In Figure 6 C,E,G, the paired-pulse ratio is shown as a function of the time interval 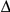.

Finally, to test wether receptor trafficking can have a larger impact on the number of open AMPARs during high synaptic activity, (10 pulses at 20 Hz), we simulate up to 10 pulses at 20 Hz. For the JS scheme, after ten pulses, the differences between diffusing or stationary AMPARs is about 3.8%, however the difference increases to 12.7% for the MN scheme (receptors are not bound to transmembrane AMPARs regulatory proteins) and 12.5% in the RL scheme. Figures 6 D,F,H display the increase in the number of desensitized receptors as a function of time. These results show that perisynaptic receptors also become desensitized, and are subsequently exchanged with AMPARs at the PSD, but they do not contribute to synaptic transmission. During the 20 Hz stimulation, some perisynaptic receptors do contribute to replenishment of the PSD receptor pool, which facilitate synaptic transmission. We further vary reservoir size, first considering a reservoir with 50% of its size located outside the cleft and subsequently one with an extra-cleft three times larger, see Figures 6 D,F,H. Because the radius of the synapse is about 200 nm, receptors have time to diffuse to the PSD. Interestingly, increasing the reservoir size by adding an extra-cleft region can contribute to the synaptic recovery of respectively 23% and 29% after ten pulses (Fig. 6 F). We conclude that AMPAR trafficking can balance freely diffusing desensitized receptors in small synapses, and this effect is controlled by the size of the reservoir, modeling the perisynaptic space.

### Synaptic transmission is depressed by fast spiking but can be rescued by reduction of vesicle release probability

**Figure 7:**
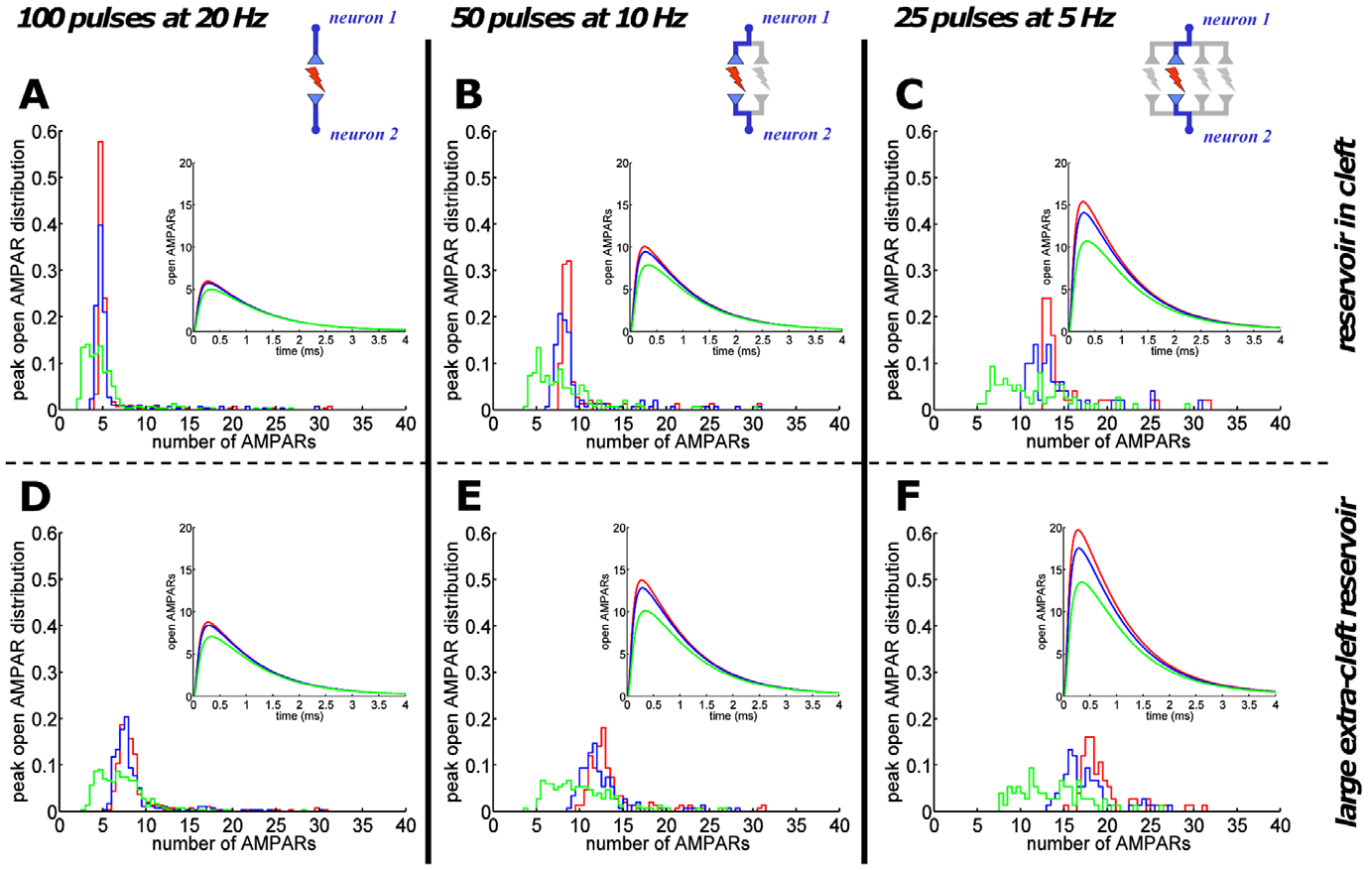
Recovery from postsynaptic depression by spike decorrelation and reservoir enlargement. A spike train at a single synaptic connection can lead to strong postsynaptic depression. The normalized distributions of the maximal number of open AMPARs (for the MN scheme) per pulse at a single participating synapse are shown for different stimulation intensities. Insets: averaged spike-to-spike time course of AMPAR openings. During a single simulated Poissonian spike train, one vesicle was released per pulse where the release sites were 1) clustered at the AZ center (red), 2) uniformly distributed over PSD (blue), 3) uniformly distributed over the cleft (green). Enlarging the AMPAR reservoir from intra-cleft only (A–C) to an additional extra-cleft one of fourfold size (D–F) increases the averaged synaptic response.

When a train of action potentials is fired at high frequency, a fraction of AMPARs will not contribute to the synaptic current due to desensitization. To investigate such effect, we estimate the number of open AMPARs following a single spike embedded in a spike train. Due to the long duration of the spike trains, receptor trafficking can be expected to play a role, thus we consider two different reservoir sizes. In the first case, the reservoir is located inside the cleft only. For 100 pulses at 20 Hz, (Fig. 7 A) the average maximal number of open AMPARs is around 6 (out of 130 AMPARs inside the cleft). For 50 pulses at 10 Hz, the maximal number of open AMPARs is 10, and for 25 pulses at 5 Hz, it is 15. These numbers do not differ for stationary receptors (data not shown) as the fraction of non-desensitized receptors inside the reservoir is very small. In the second case, we increased the reservoir fourfold corresponding to an additional 120 AMPARs in the extra-cleft reservoir. For spike train frequencies (number of pulses) of 20 Hz (100), 10 Hz (50), 5 Hz (25), the average maximal open AMPARs are 9, 14, 20. The effect of doubling reservoir size is presented in Section 5 in Text S1. We conclude that desensitization can drastically affect the synaptic response during a spike train. If the spike frequency is not too high (less than 10 Hz), this depression can be partially compensated by a large AMPAR reservoir, the size of which is however not arbitrary. For a vesicle release probability close to one, a 20 Hz or higher spike train leads to a reduction of one fifth of the synaptic current.

A low release probability such as 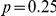 together with a large extra-cleft reservoir would restore up to two thirds of the maximal postsynaptic current response (shown in Figs. 4 E,H). Interestingly, although a low release probability (around 0.25) would decrease the frequency at a single synapse, this effect would be compensated by a significant postsynaptic current (multiplied by 3).

## Discussion

We have presented here a computational model to estimate the postsynaptic current mediated by AMPARs. The present approach features glutamate diffusion in the synaptic cleft, AMPAR trafficking in and out of the PSD, AMPAR activation modeled by kinetic schemes, and transporters located on an astroglial sheath which can take up glutamate molecules. We have shown that changing the glial distances in a range of 20 to 40 nm affects the number of open AMPARs by at most 15%, when vesicles are released in a small centered AZ. Moreover, the synaptic current is maximal when receptors are clustered at the PSD, suggesting that PSD receptor localization plays a fundamental role for the synaptic current. Adding 50% of receptors extrasynaptically followed by a translocation to the PSD using scaffolding molecules, leads to an increase of 27% in the first step and an additional 23% in the second step, resulting in an approximate total increase of 50% of the current, suggesting that LTP can be viewed as a two-step process. Finally, AMPAR trafficking can balance freely diffusing, desensitized receptors in small synapses, and this effect is controlled by the size of the perisynaptic space, which maintains a specific density of receptors. Thus for certain synapses with a large perisynaptic region, where most of the surface extrudes from the synaptic cleft, synaptic desensitization can be partially compensated by AMPAR trafficking for a spiking frequency less than 10 Hz, while a release probability bigger than 0.2 extends this property to 50 Hz.

### The perisynaptic microdomain shapes the postsynaptic response

How the perisynaptic microdomain can control the amount of AMPAR at synapses? The postsynaptic bouton is organized in multiple compartments such as the PSD that concentrates scaffolding molecules, the peri- and extrasynaptic space, and the dendritic spine that isolates the head from the dendritic shaft. The amount of receptors in the dendrite is about 10 times higher than at synapses [32]. Were all receptors free to move at equilibrium between the dendrite and dendritic spines, synaptic specificity would be lost, and this would imply that the synaptic weight would only be controlled by scaffolding molecules, which are found in large excess (compared to bound AMPARs) at the PSD [33].

Postsynaptic AMPAR density depends on surface trafficking [10], [13], but receptors can also be regulated by endo- and exocytic pathways [34], [35]. This recycling mechanism is a source of AMPAR fluctuation. Indeed, blocking locally endocytosis or preventing recycling endosomal transport abolishes LTP induction in spines [35], thus AMPARs are transported from recycling endosomes back into the spine to prevent them from escaping the spine. Actually, AMPARs undergo continuous recycling by endo- and exocytosis [36]–[38]. Moreover, preventing endocytosis by uncoupling the PSD from the endocytotic zone [39] leads to a decrease in the number of AMPARs in a time scale of minutes. This result shows that local endocytosis can balance fast lateral diffusion [40]. In our work, we use the recycling concept to define a reservoir compartment where receptors can only be exchanged with the PSD. We fix the number of receptors in this reservoir and we assume that this number is maintained at equilibrium by endo/exocytosis or exchanged due to surface membrane diffusion. The reservoir is a source of AMPARs, isolated from the dendrite. Would receptors traffic continuously, in order to maintain a local increase in the concentration, a barrier should exist to prevent synaptic receptors to equilibrate with the rest of the dendrite. This barrier could either be physical, due to the spine shape or dynamic, made up by the exo-and endocytosis machinery [40], [41].

**Figure 8:**
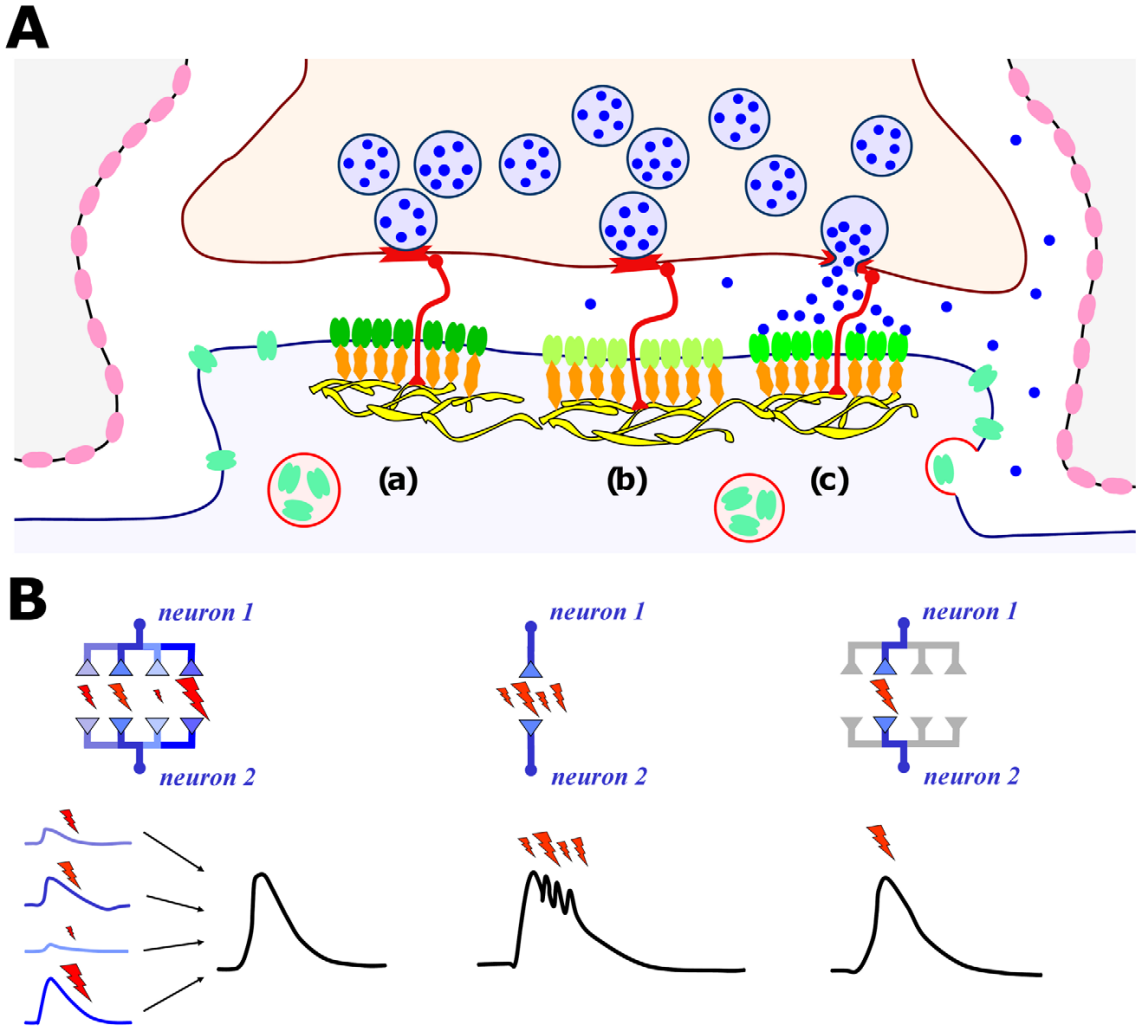
Summary of the release site-receptor alignment at simple and multiple synaptic boutons. (A) Synapse model in which clusters of AMPARs (a, b, c) are co-localized with release sites of vesicle fusion. (B) Reliable neuron-to-neuron communication can result from three signaling modes: spatial integration (over several synaptic contacts), time integration (over several bursts at a single synapse) or distributed signaling (at robust synaptic connections, see text.

As shown in Figure 5 , increasing the number of AMPARs in the perisynaptic microdomain itself leads to an increase in the number of open AMPARs. In that case, because the number of receptors at the PSD is changed, we conclude that regulating the perisynaptic size can be viewed as a form of plasticity induced by geometrical remodeling of the spine and independent of additional scaffolding molecules. In this respect, the reservoir plays a fundamental role. Furthermore, when receptors finally cluster at the PSD, a further increase in the current amplitude is achieved (Fig. 5). This suggests that synaptic plasticity may occur in two distinct stages: in a first step, receptors are just inserted and free to move in the reservoir, while in the second, they enter the PSD where they remain clustered. We conclude that increasing the number of scaffolding molecules will change the equilibrium between the PSD and the reservoir, leading to a stronger clustering of receptors and hence an increase of synaptic current (Fig. 8). Finally, it would be interesting to know what exactly determines the perisynaptic size and how the number of AMPARs is maintained there: is the dendritic spine head the location of the perisynaptic microdomain where diffusion is regulated by the thin neck? It was indeed shown that the spine neck can regulate intracellular calcium [42], [43] and receptor trafficking [31].

In the past decade, it was shown that the number of synaptic AMPARs [1], [13], [14] is not fixed but changes due to lateral diffusion and endocytotic recycling [35], [40]. It is conceivable that recycling can change the number of receptors at the PSD and thus affect the amplitude of the synaptic current. To quantify such an effect, we simulated spikes on a time scale of hundreds of milliseconds (Fig. 6) and found that in a paired pulse protocol, the fluctuation of current amplitude due to receptor trafficking was less than 5% (Fig. 6 A). However, it has recently been suggested [17] that receptor trafficking can participate functionally in synaptic transmission by significantly increasing the number of potentially available receptors and thus replacing desensitized ones. We find here that such effect can only be significant after several efficient vesicular release events, triggered by a number of spikes (at least 6 to 7), leading to a 30% recovery for large perisynaptic microdomains. During 300 ms (Fig. 6 C) of unhindered diffusion across the PSD, 70% of the moving receptors can be replaced by undesensitized extrasynaptic AMPARs. However, this result is an overestimation because in vivo, presynaptic depression will prevent vesicle release and thus provides time for the receptors to recover. Finally, recent findings suggest that the PSD undergoes constant remodeling [30], and we suggest here that these changes may affect the number of scaffolding molecules, the size and shape of the PSD, and the perisynaptic size.

### Synaptic strength depends on release site-receptor alignment and synaptic micodomains

A drastic impact of release site localization on the number of open AMPARs has been already shown in [5], [6], [44]. We confirm (Fig. 2B) that release site positioning to the periphery (ectopic release [45]) can decrease the amount of open AMPAR by 50%. Vesicles are released at the active zone [46]–[49] and as shown in Figure 4, apposition of the postsynaptic receptors to the release site is a fundamental requirement for an optimal synaptic transmission in which the mean number of open AMPARs is high, but the variance is low. It is still unclear how this apposition can be achieved, but adhesion molecules such as neuroligin/neurexin may play a major role [18]. Indeed, N-cadherin molecules, present in both, the pre- and postsynaptic terminal, can provide the apposition information since they interact directly with the extracellular domain of AMPARs and can influence the clustering of AMPA-receptors [18]. Moreover, scaffolding molecules can transmit, to the presynaptic terminal, the location of the PSD and AMPAR accumulation via these adhesion molecules [18]. In addition, N-cadherin was found to associate with AMPARs and regulate their trafficking in neurons [50]. Other molecules such as beta-catenin may also be involved, because ablation of beta-catenin in the postsynaptic neuron reduces the amplitude of spontaneous excitatory synaptic responses mediated by AMPARs [51]. In addition, at the presynaptic terminal, N-cadherin molecules may define the spot where vesicles should be released and regulate their clustering [52], [53]. Interestingly, impairing the adhesive activity of cadherins by deletion of 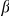-catenin or N-cadherin was found to reduce the number of reserved pool synaptic vesicles in the presynaptic terminal, resulting in an enhanced synaptic depression during repetitive stimulation [54]. In addition, the neurexin/neuroligin complex has been shown to modulate presynaptic release probability.

The apposition of active zone and PSD seems to be fundamental for synaptic transmission: It allows vesicles to be released at a favorable location relative to the localization of AMPAR clusters such that the probability of activation by glutamate is maximal. In addition, recent evidence [55] indicates that a released vesicle can induce docking of new vesicles to the same spot via a direct actin wire and favor an active zone with a finite number of hot spots for vesicle fusion. Another possibility is that docked vesicles move into the active zone by diffusion and only fuse at a finite number of distinguished locations apposed to AMPAR clusters. However, after 4 to 5 pulses, the probability that a vesicle is released at the same spot should decrease rapidly and thus an efficient release should occur at a different location. This scenario suggests that to sustain high-frequency activity in a single synapse, the active zone contains several hot spots for vesicle docking. Interestingly, various AMPAR clusters have already been reported [56]. We conclude that the apposition of active zone to PSD is fundamental for an optimal synaptic transmission and should be very well controlled at the molecular level. A reduction of the AMPAR current can result from receptor de-clustering or enlarging the active zone or both altogether.

### Receptor clustering modulates evoked synaptic transmission and miniature events

We have shown in Figure 4 that apposition of AMPARs and release sites reduces the variance and increases the mean of the synaptic current in comparison to the extreme case where release sites are uniformly distributed. Because adding extrasynaptic receptors (Fig. 5) increases the synaptic current, we propose that this represents a first step in the LTP process. In a second step, receptors can move by diffusion inside the PSD, where scaffolding molecules in excess [33] can bind them. Increased number of scaffolding molecules will prolong the resident time of the receptor at the PSD [31], [57].

Interestingly, the possibility to obtain LTP in PSD95 knockout mice [58] can be interpreted within this model as the aforementioned first step leading to more AMPARs in the reservoir which may even result in an increase of PSD receptors. We predict that the postsynaptic response should be quite unreliable. However, as scaffolding molecules are being expressed and localized at the PSD, the CV of the current should decay. Actually, increasing the number of scaffolding molecules may be part of the development process to increase the synaptic efficacy. Conversely, a protocol that results in detaching AMPARs would lead to a decrease in the synaptic current amplitude (Fig. 4), thus reducing the detection threshold of the post synaptic neuron. Interestingly, the PSD95 KO mice can sustain LTP, and the frequency of minis is diminished while the amplitude of synaptic current is not affected [1], [58]. From our analysis, we can now postulate that a synapse should contain multiple structures where vesicular fusion spots are apposed to one or several clusters of AMPARs (Fig. 8). Disrupting scaffolding molecules should affect some of these subsynaptic structures, while others remain functional. In that case, the postsynaptic detection threshold will decrease, implying a reduction in the postsynaptic frequency, while the remaining sub-synaptic structures would still generate an EPSC of an amplitude comparable to the control case. Overexpressing PSD95 could lead to the formation of new AMPAR clusters and the formation of additional sub-synaptic structures [1], [58].

### Efficient transmission for spiking neurons requires several depressing synaptic boutons

Vesicular release is not a reliable process [2], [59]: Only sometimes a spike triggers a vesicle release. Although this process has been well studied [60], many of the molecular details are still lacking, but for various types of neurons such as CA1-hippocampal neurons, the release probability 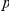 is estimated to be around 0.2. Our analysis of Figure 7 suggests that a low release probability allows to decorrelate spikes firing at 10 Hz at least. For example, in the absence of depression, a release probability of 1 at a spike train of 20 Hz would result in a postsynaptic current mediated by 6 open AMPARs, while for an unreliable synapse, i.e., with release probability of around 0.2, the current would increase threefold. Interestingly, temporal correlation leads to receptor desensitization which cannot be compensated by receptor trafficking alone (Figs. 7 A,D). We conclude that preventing vesicular release allows desensitized AMPARs to recover and provides time during which fresh receptors can enter the synapse by trafficking. Hence a release event activates much more AMPARs and thus can generate a significant EPSC. Even though this synaptic unreliability property restricts on possible spiking frequencies, it seems that fast signaling can be restored at the cellular level. Indeed, it has been shown [19] that a presynaptic neuron can have multiple connections with a postsynaptic one, from one to several (5 on average).

Although a single synapse is an unreliable device, there are several ways by which neuron-to-neuron connections can still be made: 1) reliable, in the sense that synaptic signals are actually elicited, and 2) robust, in the sense that the resulting postsynaptic current is significant and has a low variance. These ways are illustrated in Figure 8B: one way is to integrate (in space) and hence average a given signal over several unreliable synapses that produce highly variable postsynaptic currents. A second possibility is to replace a single spike by a spike burst, which can increase release probability (hence reliability), such that the signal integration (in time) takes place over the postsynaptic currents of every elicited event in the burst. This scenario is equivalent to releasing a large number of vesicles at the same synapse. A third possibility is to distribute the signal over several robust synaptic connections and to reduce the release probability (e.g., for 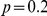 and 5 synaptic connections). As discussed above, synaptic robustness can be achieved by apposition of receptors and release sites. For this mechanism to work, i.e., to bring vesicles at the designated sites, a certain minimal time scale may actually be required. While the first two scenarios rely on increasing synaptic activity and therefore require more cellular energy, the third one relies on a local and selective activity. It is possible that different populations of neurons use one of these different possibilities. However, the third scenario of neuronal connection raises several questions: can the release probability be dependent on the number of synaptic connections? Are sister synapses between two neurons really independent? It is quite surprising that synaptic unreliability [61] can have such an effect on neuronal transmission.

To conclude, we summarize the main sources of synaptic fluctuations which contribute to synaptic unreliability: 1) synaptic geometry, 2) location of vesicle fusion, 3) apposition of release sites and AMPAR clusters, 4) low release probability. In the present analysis, we show that presynaptic depression leads to decoupling of spikes and hence to a higher synaptic current. Interestingly, multi-synaptic connections are likely fundamental to achieve a robust cellular transmission. In that context, we suggest that unreliable synapses allow actually a reliable synaptic transmission at high frequency.

## Materials and Methods

### Electrophysiology

Experiments were carried out according to the guidelines of the European Community Council Directives of November 24th 1986 (86/609/EEC) and approved by the ethical committee of Paris 1, agreement number 2009-0014. C57Bl6 mice (wildtype (wt)) were supplied by Charles River, L'Arbresle, France. For all analyses, mice of both genders and were used (P16–P25). Acute transverse hippocampal slices (300–400 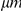) were prepared as previously described [32]. Slices were maintained at room temperature in a storage chamber that was perfused with an artificial cerebrospinal fluid (ACSF) containing (in mM): 119 NaCl, 2.5 KCl, 2.5 CaCl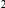, 1.3 MgSO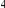, 1 NaH
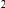
PO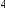, 26.2 NaHCO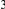 and 11 glucose, saturated with 95% O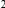 and 5% CO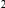, for at least one hour prior to recording. Slices were transferred to a submerged recording chamber mounted on an Olympus BX51WI microscope equipped for infra red-differential interference (IR-DIC) microscopy and were perfused with ACSF at a rate of 1.5 ml/min at room temperature. All experiments were performed in the presence of picrotoxin (100 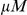) and a cut was made between CA1 and CA3 to prevent the propagation of epileptiform activity. Somatic whole-cell recordings were obtained from visually identified CA1 pyramidal cells and stratum radiatum astrocytes, using 5–10 M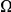 glass pipettes filled with either (in mM): 115 CsMeSO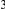, 20 CsCl, 10 HEPES, 2.5 MgCl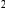, 4 Na
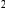
ATP, 0.4 NaGTP, 10 Na-phosphocreatine, 0.6 EGTA, 0.1 spermine, 5 QX314 (pH 7.2, 280 mOsm). Miniature excitatory postsynaptic currents (mEPSCs) were recorded at −70 mV in the presence of 0.5 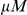 TTX. Evoked postsynaptic responses were induced by stimulating Schaffer collaterals (0.1 Hz) in CA1 stratum radiatum with ACSF filled glass pipettes. Stimulus artifacts were blanked in sample traces. Recordings were acquired with Axopatch-1D amplifiers (Molecular Devices, USA), digitized at 10 kHz, filtered at 2 kHz, stored and analyzed on computer using Pclamp9 and Clampfit9 softwares (Molecular Devices, USA). All data are expressed as mean 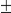 SEM. Picrotoxin was obtained from Sigma, all other chemicals from Tocris.

### Simulation

We describe a simulation and modeling approach for the synaptic cleft. All programs were written in MATLAB and C. Multiple Monte Carlo simulations were performed for a discretization time step of 0.5 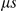. The default values for all parameters are listed in Table 1 unless stated otherwise.

**Table 1:** Simulation parameters.

Length of extrasynaptic space (pre- plus postsynaptic cylinders)	1 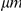
Cleft height	20 nm
Distance of the glial sheath from the synaptic cylinder surfaces	40 nm
Diameter of the PSD	200 nm [33]
Diameter of the cleft	400 nm [33]
Vesicle content	3000 glutamate molecules
Glutamate diffusion constant	0.2 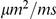 [7]
AMPARs on the PSD	100
AMPARs in the intra-cleft reservoir	30
AMPAR diffusion constant	0.1 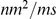 [17]
Transporter densities on the glial sheath	2,500 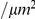 to 10,000 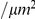
Time step size 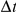	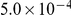 ms

Default values of the simulation parameters (unless explicitly stated otherwise).

#### Synapse geometry and functionality

The presynaptic and postsynaptic elements were modeled as two coaxial cylinders of length 0.5 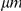 each and 400 nm diameter. The distance between these cylinders represents the synaptic cleft height (20 nm). The glial sheet was designed as coaxial cylindrical surface surrounding the pre and postsynaptic cylinders at a distance of 40 nm. The postsynaptic density was defined as a circular area of 200 nm in diameter, centered on the surface of the postsynaptic cylinder (see Fig. 1).

#### Vesicle release

Vesicle release sites were generally placed on the surface of the presynaptic cylinder. A single vesicle contains 3000 glutamate molecules, which, upon vesicle fusion, were all released at a single point and in a single time step.

#### Glutamate diffusion

Upon release, glutamate could diffuse freely with a diffusion constant of 0.2 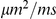 [7], [62]. As shown in http://arxiv.org/abs/1104.1090, variation in the glutamate diffusion constant does not affect the probability of glutamate to bind before exiting the synaptic cleft. It only affects the kinetics, however, this binding kinetics is already extremely fast, (of the order of 100 mu s), much faster than any other processes of bindings (time of ms). Thus any changes in 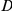 (which can be multiplied by 2) do not affect much the synaptic current. Glutamate trajectories were simulated according to Brownian dynamics. Upon hitting a membrane surface, they were specularly reflected (or bound on transporters, see below). Upon reaching a distance of 0.5 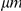 away from the cleft center, a trajectory was terminated.

#### AMPA-Receptors

AMPA-Receptors were placed in two areas: on the PSD and in the reservoir. The reservoir contains an intra-cleft part, i.e., the cleft-facing disk of the postsynaptic cylinder without the PSD, and an extra-cleft part, i.e., area on the lateral postsynaptic cylinder surface (see Fig. 1). Unless stated otherwise, at simulation start, AMPARs were uniformly distributed in the intra- and extra-cleft areas such that the ratio of densities of PSD-AMPARs to reservoir-AMPARs was 10∶1 where 100 AMPARs were placed on the PSD. AMPARs trafficked in these areas at a diffusion constant of 0.1 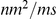 [17]. AMPAR trajectories were simulated by Brownian dynamics. Due to AMPARs binding to PSD scaffolding molecules and confinement in micro-domains on the PSD, AMPARs accumulate at a higher concentration on the PSD compared to the reservoir. The mean AMPAR densities on PSD and reservoir were maintained constant by free trafficking of AMPARs from the reservoir into the PSD. To simulate the PSD corral, the passage from the PSD into the reservoir is successful only one every tenth attempts and are otherwise the AMPAR is reflected at the PSD boundary (see Section 7.2.3 in Text S1 for details). At the outer boundary of the reservoir, AMPARs sent back into the reservoir. Internal states of AMPARs were modeled using the Markov schemes by Jonas-Sakmann [24] (which is called JS scheme in this paper), by Milstein-Nicoll [25] (called MN scheme), and by Raghavachari-Lisman [21] (called RL scheme). We refer to Section 6 in Text S1 for the JS, MN, and RL schemes, and a comparison of them. The random fluctuations of the internal states of AMPARs were modeled as fluctuations of the number of glutamate molecules near the receptor excluding fluctuations of the Markov chain. A small circular area was associated to every AMPAR, where the internal state dynamics was inferred from the number of glutamate molecules hitting this area per time step. Glutamate molecules hitting this area were then reflected and glutamate binding was neglected, see Section 7 in Text S1. The internal states of AMPARs located outside the cleft were not affected by hitting glutamate.

#### Glial transporters

The glial sheath was uniformly covered with glutamate transporters which were located on an equally-spaced square grid at different densities ranging from 2,500 to 10,000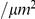. Glial glutamate transporters can bind glutamate molecules and internalize them into the glia. To model these kinetics, we used a Markov scheme [7] (see Text S1). A small circular area was associated to every transporter and every glutamate molecule hitting this area was either specularly reflected or bound such that the binding rate of the Markov scheme was assumed. Depending on the state transitions of the scheme, the glutamate molecule was either unbound, i.e., reinserted into the extrasynaptic space, or internalized, i.e., taken out of the simulation. See Section 7 in Text S1 for a complete description of the simulation procedure.

## Supporting Information

Text S1 **Presents the following information: In Section 1, the derivation of the formula for the PSD-reservoir receptor exchange rates.** In Section 2, additional data for Figure 2 regarding synaptic geometry with the JS and MN AMPAR models, and for uniformly distributed release sites. In Section 3, additional data for Figure 3 regarding glutamate spread for doubled glutamate diffusion constant. In Section 4, further comments on AMPAR trafficking and synaptic transmission. In Section 5, comments on the effect of reservoir size on pulse trains. In Section 6, JS, MN, and RL AMPAR kinetic models are compared. Section 7 provides a detailed simulation analysis and description of algorithms.
(PDF)

## References

[1] Elias GM, Nicoll RA (2007). Synaptic tra_cking of glutamate receptors by MAGUK scaffolding proteins.. Trends Cell Biol.

[2] Zucker RS (2005). Minis: whence and wherefore?. Neuron.

[3] Rusakov DA (2001). The role of perisynaptic glial sheaths in glutamate spillover and extracellular Ca(2+) depletion.. Biophys J.

[4] Holmes WR (1995). Modeling the effect of glutamate diffusion and uptake on NMDA and non-NMDA receptor saturation.. Biophys J.

[5] Barbour B (2001). An evaluation of synapse independence.. J Neurosci.

[6] Xie X, Liaw JS, Baudry M, Berger TW (1997). Novel expression mechanism for synaptic potentiation: alignment of presynaptic release site and postsynaptic receptor.. Proc Natl Acad Sci USA.

[7] Franks KM, Stevens TM, Sejnowski TJ (2003). Independent sources of quantal variability at single glutamatergic synapses.. J Neur.

[8] Lisman JE, Raghavachari S, Tsien RW (2007). The sequence of events that underlie quantal transmission at central glutamatergic synapses.. Nat Rev Neurosci.

[9] Conti R, Lisman J (2003). The high variance of AMPA receptor- and NMDA receptor-mediated responses at single hippocampal synapses: evidence for multiquantal release.. Proc Natl Acad Sci USA.

[10] Chen L, Chetkovich DM, Petralia RS, Sweeney NT, Kawasaki Y (2000). Stargazin regulates synaptic targeting of AMPA receptors by two distinct mechanisms.. Nature.

[11] Bredt DS, Nicoll RA (2003). AMPA receptor trafficking at excitatory synampses.. Neuron.

[12] Borgdorff AJ, Choquet D (2002). Regulation of AMPA receptor lateral movements.. Nature.

[13] Choquet D, Triller A (2003). The role of receptor diffusion in the organization of the postsynaptic membrane.. Nat Rev Neurosci.

[14] Triller A, Choquet D (2005). Surface trafficking of receptors between synaptic and extrasynaptic membranes: and yet they do move!. Trends Neurosci.

[15] Shi SH, Hayashi Y, Petralia RS, Zaman SH, Wenthold RJ (1999). Rapid spine delivery and redistribution of AMPA receptors after synaptic NMDA receptor activation.. Science.

[16] Malenka RC, Nicoll RA (1999). Long-term potentiation–a decade of progress?. Science.

[17] Heine M, Groc L, Frischknecht R, Béique JC, Lounis B (2008). Surface mobility of postsynaptic AMPARs tunes synaptic transmission.. Science.

[18] Gerrow K, El-Husseini A (2007). Receptors look outward: revealing signals that bring excitation to synapses.. Science.

[19] Silver RA, Lubke J, Sakmann B, Feldmeyer D (2003). High-probability uniquantal transmission at excitatory synapses in barrel cortex.. Science.

[20] Wahl LM, Pouzat C, Stratford KJ (1996). Monte carlo simulation of fast excitatory transmission at a hippocampal synapse.. J Neurophysiol.

[21] Raghavachari S, Lisman JE (2004). Properties of quantal transmission at ca1 synapses.. J Neurophysiol.

[22] Franks KM, Bartol TM, Sejnowski TJ (2002). A monte carlo model reveals independent signaling at central glutamatergic synapses.. Biophys J.

[23] Bergles DE, Jahr CE (1997). Synaptic activation of glutamate transporters in hippocampal astrocytes.. Neuron.

[24] Jonas P, Major G, Sakmann B (1993). Quantal components of unitary EPSCs at the mossy fibre synapse on ca3 pyramidal cells of rat hippocampus.. J Physiol.

[25] Milstein AD, Zhou W, Karimzadegan S, Bredt DS, Nicoll RA (2007). Tarp subtypes differentially and dose-dependently control synaptic AMPA receptor gating.. Neuron.

[26] Markus EJ, Petit TL, LeBoutillier JC (1987). Synaptic structural changes during development and aging.. Brain Res.

[27] Jiang ML, Han TZ, Yang DW, Chen MX (2003). Morphological alteration of the hippocampal synapses in rats prenatally exposed to magnetic resonance imaging magnetic fields.. Acta Physiologica Sinica.

[28] Melone M, Bellesi M, Conti F (2009). Synaptic localization of glt-1a in the rat somatic sensory cortex.. Glia.

[29] Ventura R, Harris KM (1999). Three-dimensional relationships between hippocampal synapses and astrocytes.. J Neurosci.

[30] Blanpied TA, Kerr JM, Ehlers MD (2008). Structural plasticity with preserved topology in the postsynaptic protein network.. Proc Natl Acad Sci USA.

[31] Holcman D, Triller A (2006). Modeling synaptic dynamics driven by receptor lateral diffusion.. Biophys J.

[32] Rouach N, Byrd K, Petralia RS, Elias GM, Adesnik H (2005). TARP gamma-8 controls hippocampal AMPA receptor number, distribution and synaptic plasticity.. Nat Neurosci.

[33] Sheng M, Hoogenraad CC (2007). The postsynaptic architecture of excitatory synapses: a more quantitative view.. Annu Rev Biochem.

[34] Park M, Salgado JM, Ostroff L, Helton TD, Robinson CG (2006). Plasticityinduced growth of dendritic spines by exocytic trafficking from recycling endosomes.. Neuron.

[35] Park M, Penick EC, Edwards JG, Kauer JA, Ehlers MD (2004). Recycling endosomes supply AMPA receptors for LTP.. Science.

[36] Carroll RC, Lissin DV, von Zastrow M, Nicoll R, Malenka RC (1999). Rapid redistribution of glutamate receptors contributes to long-term depression in hippocampal cultures.. Nat Neurosci.

[37] Luscher C, Nicoll RA, Malenka RC, Muller D (2000). Synaptic plasticity and dynamic modulation of the postsynaptic membrane.. Nat Neurosci.

[38] Ehlers MD (2000). Reinsertion or degradation of AMPA receptors determined by activity-dependent endocytic sorting.. Neuron.

[39] Lu J, Helton TD, Blanpied TA, Racz B, Newpher TM (2007). Postsynapticpositioning of endocytic zones and AMPA receptor cycling by physical coupling of dynamin-3 to homer.. Neuron.

[40] Newpher TM, Ehlers MD (2008). Glutamate receptor dynamics in dendritic microdomains.. Neuron.

[41] Ashby MC, Maier SR, Nishimune A, Henley JM (2006). Lateral diffusion drivesconstitutive exchange of AMPA receptors at dendritic spines and is regulated by spine morphology.. J Neurosci.

[42] Korkotian E, Holcman D, Segal M (2004). Dynamic regulation of spine-dendrite coupling in cultured hippocampal neurons.. Eur J Neurosci.

[43] Biess A, Korkotian E, Holcman D (2007). Diffusion in a dendritic spine: the role of geometry.. Phys Rev E.

[44] Ventriglia F, Di Maio V (2002). Stochastic uctuations of the synaptic function.. Biosystems.

[45] Matsui K, Jahr CE (2003). Ectopic release of synaptic vesicles.. Neuron.

[46] Bourne JN, Harris KM (2008). Balancing structure and function at hippocampal dendritic spines.. Annu Rev Neurosci.

[47] Sudhof TC (2004). The synaptic vesicle cycle.. Annu Rev Neurosci.

[48] Bloom O, Evergren E, Tomilin N, Kjaerulff O, Low P (2003). Colocalization of synapsin and actin during synaptic vesicle recycling.. J Cell Biol.

[49] Cingolani LA, Goda Y (2008). Actin in action: the interplay between the actin cytoskeleton and synaptic efficacy.. Nat Rev Neurosci.

[50] Nuriya M, Huganir RL (2006). Regulation of AMPA receptor trafficking by Ncadherin.. J Neurochem.

[51] Okuda T, Yu LM, Cingolani LA, Kemler R, Goda Y (2007). beta-catenin regulates excitatory postsynaptic strength at hippocampal synapses.. Proc Natl Acad Sci USA.

[52] Gottmann K (2008). Transsynaptic modulation of the synaptic vesicle cycle by celladhesion molecules.. J Neurosci Res.

[53] Bozdagi O, Valcin M, Poskanzer K, Tanaka H, Benson DL (2004). Temporally distinct demands for classic cadherins in synapse formation and maturation.. Mol Cell Neurosci.

[54] Birchmeier W, Lu B, Reichardt LF (2003). Role of beta-catenin in synaptic vesicle localization and presynaptic assembly.. Neuron.

[55] Siksou L, Rostaing P, Lechaire JP, Boudier T, Ohtsuka T (2007). Threedimensional architecture of presynaptic terminal cytomatrix.. J Neurosci.

[56] Masugi-Tokita M, Tarusawa E, Watanabe M, Molna'r E, Fujimoto K (2007). Number and density of AMPA receptors in individual synapses in the rat cerebellum as revealed by sds-digested freeze-fracture replica labeling.. J Neurosci.

[57] Taia A, Holcman D (2007). Dwell time of a brownian molecule in a microdomainwith traps and a small hole on the boundary.. J Chem Phys.

[58] Elias GM, Funke L, Stein V, Grant SG, Bredt D (2006). Synapse-specific and developmentally regulated targeting of AMPA receptors by a family of MAGUK scaffolding proteins.. Neuron.

[59] Zucker RS (1989). Short-term synaptic plasticity.. Annu Rev Neurosci.

[60] Schneggenburger R, Sakaba T, Neher E (2002). Vesicle pools and short-term synaptic depression: lessons from a large synapse.. Trends Neurosci.

[61] Tsodyks MV, Markram H (1997). The neural code between neocortical pyramidal neurons depends on neurotransmitter release probability.. Proc Natl Acad Sci USA.

[62] Nielsen TA, Digregorio DA, Silver RA (2005). Modulation of glutamate mobility reveals the mechanism underlying slow-rising ampar epscs and the diffusion coe_cient in the synaptic cleft.. Neuron.

