# Supplementary material for: Synapse Geometry and Receptor Dynamics Modulate Synaptic Strength
Source: PLoS One. 2011 Oct 3;6(10):e25122. doi: 10.1371/journal.pone.0025122 (PMC3184958; doi:10.1371/journal.pone.0025122)
Supplement: Text S1 — Presents the following information: In Section 1, the derivation of the formula for the PSD-reservoir receptor exchange rates. In Section 2, additional data for Figure 2 regarding synaptic geometry with the JS and MN AMPAR models, and for uniformly distributed release sites. In Section 3, additional data for Figure 3 regarding glutamate spread for doubled glutamate diffusion constant. In Section 4, further comments on AMPAR trafficking and synaptic transmission. In Section 5, comments on the effect of reservoir size on pulse trains. In Section 6, JS, MN, and RL AMPAR kinetic models are compared. Section 7 provides a detailed simulation analysis and description of algorithms. (PDF) [file pone.0025122.s001.pdf]

SUPPORTING TEXT S1

Synapse geometry and receptor dynamics modulate synaptic strength

D. Freche[1], U. Pannasch[2], N. Rouach[2], D. Holcman[3]

Contents

1 Formula for the PSD-reservoir receptor exchange rates **2**

2 Figure 2: Synaptic geometry with the JS and MN AMPAR models, and for uniformly distributed release sites **3**

3 Figure 3: Glutamate spread for doubled glutamate diffusion constant **4**

4 AMPAR trafficking and synaptic transmission: Further comments **5**

5 Effect of reservoir size on pulse trains: Further comments **6**

6 Comparison of the JS, MN, and RL AMPAR kinetic models **8**

7 Simulation analysis and description of algorithms **10**

7.1 Simulation of glutamate diffusion 10

7.2 Simulation of AMPA-receptors and glial transporters 10

7.2.1 Simulation of AMPAR activation 11

7.2.2 Simulation of glial transporters 11

7.2.3 Simulation of AMPAR trafficking and maintaining prescribed AMPAR concentrations in PSD and reservoir 12

7.2.4 The local glutamate concentration 12

7.2.5 Comparing the Brownian dynamics simulation with a differential equation solver . . . 14

[1]Department of Mathematics, Weizmann Institute of Science, Rehovot 76100, Israel.

[2]INSERM U 840 College de France, 11 Place Marcelin Berthelot, 75005.

[3]Department of Computational Biology, Ecole Normale Supérieure, 46 rue d'Ulm 75005 Paris, France, and Department of Applied Mathematics, Tel Aviv University, Tel Aviv 69978, Israel. Email: holcman@biologie.ens.fr

1 Formula for the PSD-reservoir receptor exchange rates

To calculate the mean receptor exchange rate between the PSD and the surrounding reservoir (perisynaptic area), we approximate the PSD by a disk of radius R_{PSD} and the reservoir by a surrounding annulus of outer radius R_{res}. AMPARs are uniformly distributed in the PSD and the reservoir with the same density. The outer reservoir boundary is a reflecting barrier for a trafficking receptor.

We model the motion of a receptor as Brownian, defined by the position X_t and the probability $p(t,x)$ of finding the receptor at position x at time t when it is initially in the PSD with uniform probability, i.e.,

$$p(t,x) = Pr(X_t \in x + dx | X_0 \in \text{PSD}) \tag{1}$$

satisfies

$$\partial_t p(t,x) = D_{AMPAR} \Delta_x p(t,x) \tag{2}$$

$$p(0,x) = \begin{cases} 1/(\pi R_{PSD}^2) & |x| < R_{PSD} \\ 0 & R_{PSD} < |x| < R_{res} \end{cases} \tag{3}$$

where D_{AMPAR} is the AMPAR diffusion coefficient. Reflecting boundary conditions are imposed at the boundary $\{|x| = R_{res}\}$. The solution is

$$p(t,r) = \frac{1}{\pi R_{res}^2} + \frac{2}{\pi R_{res} R_{PSD}} \sum_{n=0}^{\infty} \frac{J_1\big(j_n \frac{R_{PSD}}{R_{res}}\big)}{j_n J_0(j_n)^2} J_0\big(j_n \frac{r}{R_{res}}\big) e^{-D_{AMPAR} \frac{j_n^2}{R_{res}^2} t},$$

where $r = |x|$ is the radial distance to the center of the PSD, J_0, J_1 are the Bessel functions of the first kind of order 0 and 1, respectively, and j_n is the ascending sequence of zeros of J_1. The receptor exchange rate is obtained from the flux F_{ex} over the PSD boundary: when N_0 receptors are located on the PSD at time 0, we get

$$F_{ex} = -N_0 \int_{\text{PSD boundary}} D_{AMPAR} \, \partial_\nu p(t,x) \, .$$

and the number of receptors still in the PSD at time t is

$$N(t) = N_0 \int_{\text{PSD area}} p(t,x) dx$$

Using that the number of exchanged receptor during a time T is

$$\int_0^T F_{ex}(t) dt = N_0 - N(T) \, .$$

We obtain the formula for the mean number $N(t)$ of exchanged receptors

$$N(t) = N_0 \int_{\text{PSD}} p(t,r) dr = N_0 \frac{R_{PSD}^2}{R_{res}^2} + 4N_0 \sum_{n=0}^{\infty} \frac{J_1\big(j_n \frac{R_{PSD}}{R_{res}}\big)}{j_n J_0(j_n)^2} \, e^{-D_{AMPAR} \frac{j_n^2}{R_{res}^2} t} \, .$$

The decay term can be approximated by the first exponential for a time sufficiently long.

2 Figure 2: Synaptic geometry with the JS and MN AMPAR models, and for uniformly distributed release sites

Figure 2 is the same as Figure 2 in the main article, but also the JS scheme is used as AMPAR model. Additionaly, it shows the effect of locating all release sites only in the cleft center of the presynaptic terminal.

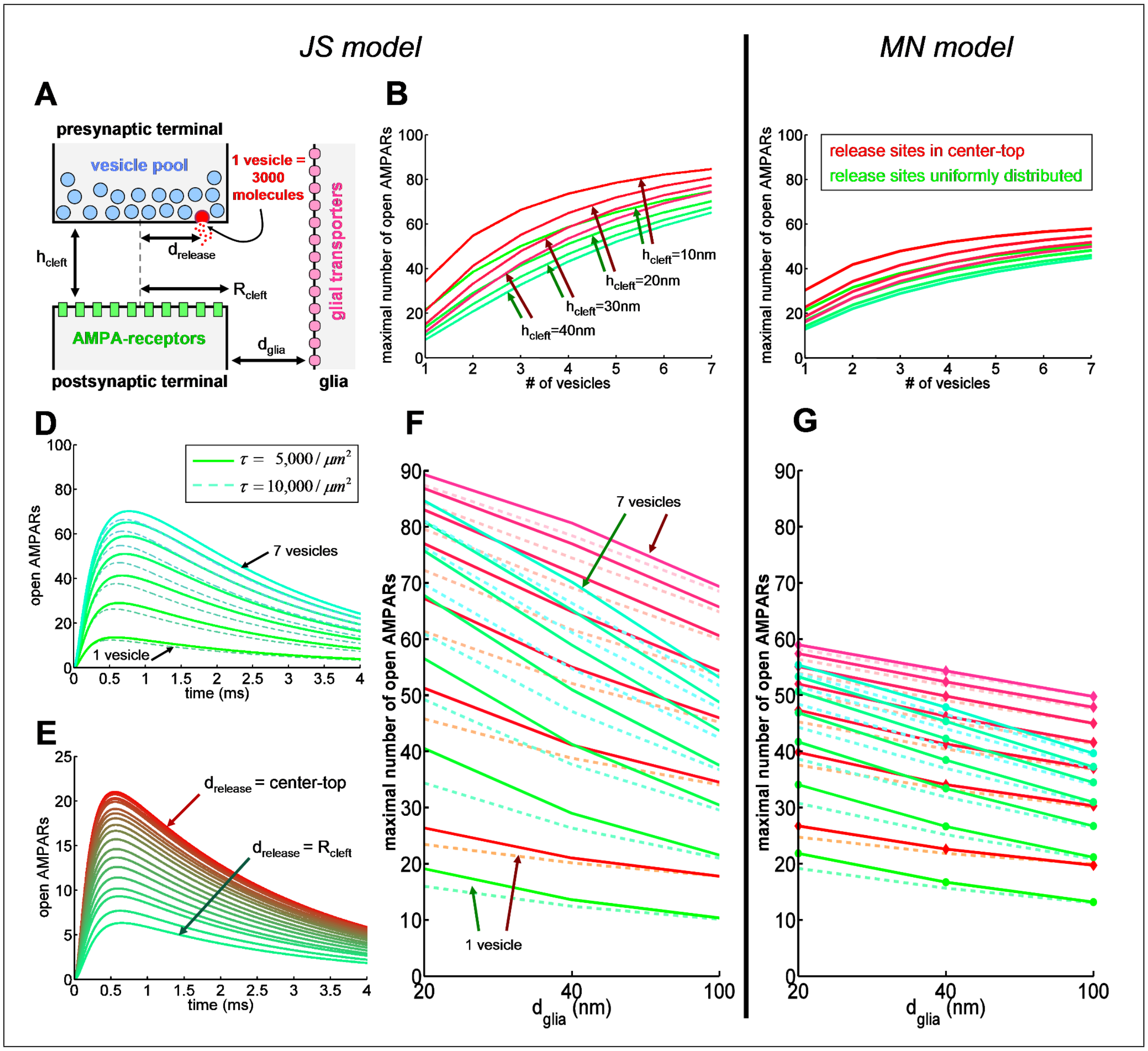

Figure 2: Dynamics of the cleft. **(A)** Schematic representation of the synaptic cleft: cleft height h_{cleft}=20nm, distance $d_{release}$=0 of vesicle release site from cleft center, distance d_{glia}=40 nm of glial sheath from cleft exit, glial transporter density τ_{transp}=$5,000/\mu m^2$, cleft radius R_{cleft}=200 nm, 130 AMPARs uniformly distributed on the postsynaptic terminal. Vesicle release sites were located either in center of the presynaptic terminal (red lines) or uniformly distributed (green lines) inside the cleft. **(B, C)** Increasing

h_{cleft}=10 nm, 20 nm, 30 nm, 40 nm decreases the number of open AMPARs. **(D)** Doubling the density of transporters τ_{transp} from $5,000$ to $10,000/\mu m^2$ has little influence on the peak open AMPAR numbers, but accelerates the time course of receptor closing. **(E)** Change in the number of open AMPARs upon varying $d_{release}$ from 0 nm to 200 nm in steps of 10 nm. The response is largest at center-top release and lowest at cleft-edge. **(F, G)** Changing d_{glia}=20nm, 40nm, 100nm affects the maximal number of open AMPARs (Released vesicles: 1 to 7, τ_{transp}=$5,000/\mu m^2$ (solid) and $10,000/\mu m^2$ (dashed)). Transporters influence transmission for small d_{glia} and low number of released vesicles.

3 Figure 3: Glutamate spread for doubled glutamate diffusion constant

Efficient removal of glutamate molecules from the extra-synaptic space is crucial to prevent spill-over and long-time desensitization of synapses. The extent of glutamate spread in the extrasynaptic space ('spreading distance') and glutamate removal by glial transporters ('clearance time') is described in Figure 3 of the main article. However, these quantities depend on the glutamate diffusion constant D in the extrasynaptic space. Extracellular diffusivity is the result of tortuousity of the extrasynaptic space. Different numbers are given in the literature; while $D = 0.2\mu m^2/ms$ [7] was used in all simulations, we here explore the effect on glutamate spreading and uptake for a doubled diffusion constant $D_{double} = 0.4\mu m^2/ms$ [8]. Figure 3 shows that the use of D_{double} increases the spreading distance by 50%, whereas the clearance time remains unaffected.

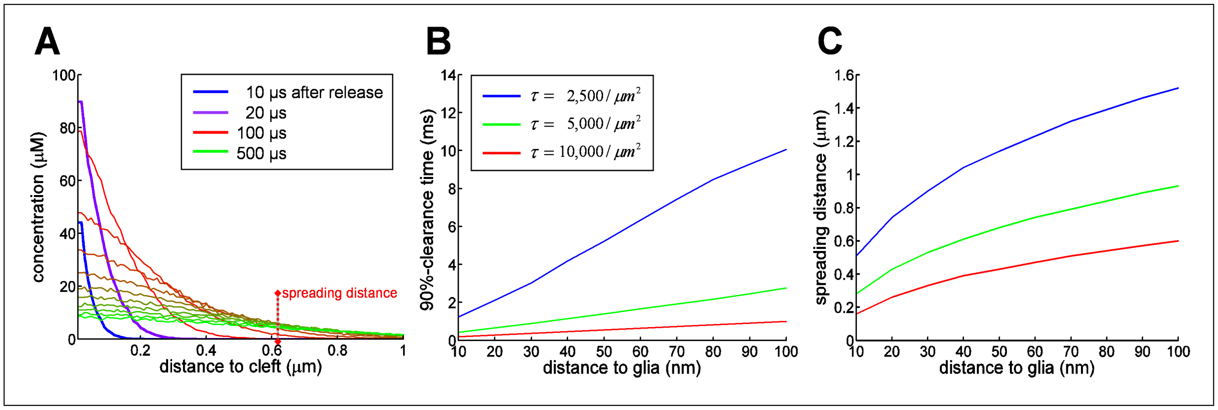

Figure 3: Glutamate dynamics in the extracellular space for a doubled diffusion constant. Effect of doubling the glutamate diffusion constant to $D = 0.4\mu\text{m}^2/\text{ms}$. **(A)** We present the glutamate density in the extra-synaptic space for different times after a vesicular release event (the glial distance is 40 nm and the transporter density is $5,000/\mu m^2$). **(B, C)** Clearance time and spreading distance for various glial-sheath distances from 10 nm to 100 nm and the transporter densities of $2,500 - 5,000 - 10,000/\mu m^2$ (synapse radius 200nm, cleft height 20nm).

4 AMPAR trafficking and synaptic transmission: Further comments

AMPAR trafficking for a lower density in the reservoir. The time course of receptor exchange strongly increases down when AMPARs can bind to scaffolding molecules on the PSD. To account for scaffolding molecules, we maintain different densities of AMPARs on the PSD and in the reservoir (see Section 7.2.3). To quantify this increase, we place 100 AMPARs on the PSD and 30, 60, 90 AMPARs, respectively, inside the reservoir adjusting its size such that the AMPAR density on PSD was ten times larger than in the reservoir. For the synaptic geometry used throughout the paper (PSD diameter 200nm, cleft diameter 400nm), this configuration imposed that a reservoir with 30 AMPARs is located completely inside the cleft, whereas those with 60 and 90 AMPARs extend to extra-cleft areas.

For a reservoir with 30 receptors at equilibrium, 23 of the 30 AMPARs are found on average in the PSD (see Fig. 4A). At 80% of equilibrium when 18 receptors (80% of 23) have reached the PSD, this happens in about 68.5ms. In other words, given 100 PSD-borne AMPARs and 30 reservoir-based AMPARs, with density ratio 10:1, 18 % of the PSD-AMPARs can be replaced in 68.5ms. For the reservoir with 60 (respectively, 90) AMPARs, 30 (38) PSD-AMPARs can be replaced in 181 ms (268 ms), see Fig. 4A. This shows the speed of the exchange dynamics of AMPARs between the reservoir and the PSD.

Paired-pulse protocol for a synapse with glial sheath. We repeated the PPR simulations of Figs. 6C,E in the main article for a synapse containing 100 AMPARs on the PSD and a reservoir at three different sizes (30, 60, and 90 AMPARs) with an area such that the PSD-to-reservoir receptor density ratio was maintained at 10:1. Furthermore, the synapse is surrounded by a glial sheath located at a distance of 40nm away, containing a transporter density of $5{,}000/\mu m^2$. Due to the smaller receptor density in the reservoir and a slower receptor exchange rate (see previous paragraph), the impact of receptor exchange on the PPR is delayed. Indeed desensitized receptors are transported out of the PSD and replaced by non depressed receptors that initially were outside the PSD. If fewer receptors are outside, it will take longer to replace the same amount of receptors compared to a synapse with a higher number of receptors outside the PSD.

Finally, compared to a glial sheath far away from the synapse (as in Figs. 6C,E in the main article), the presence of the glial sheath leads to a delayed glutamate clearance from the cleft and hence to an increased number of desensitized receptors. Thus, the PPR drops, as shown in Fig. 4B, C.

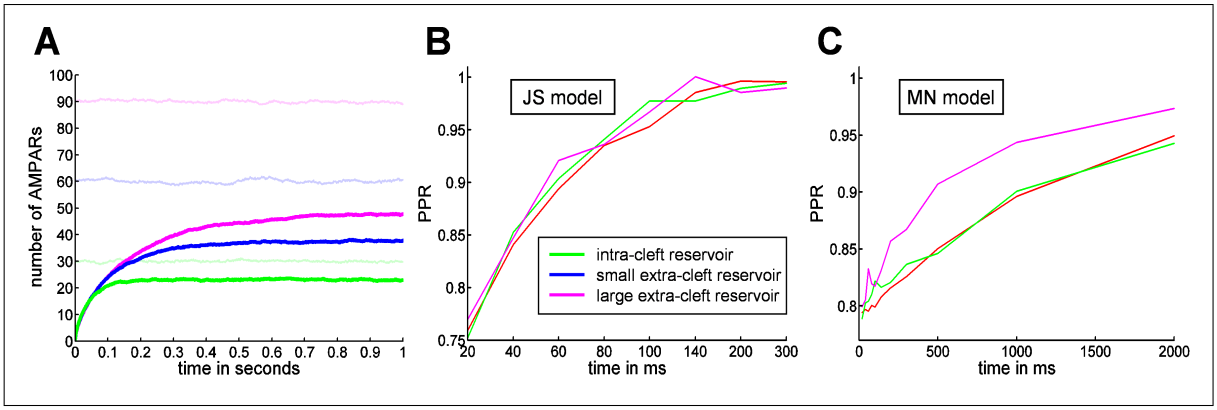

Figure 4: Effect of AMPAR trafficking, glial sheath and scaffolding molecules on synaptic transmission. (A) Maintaining a ten times larger AMPAR density on the PSD (100 AMPARs) than in the reservoir (30 (light green), 60 (light blue), 90 (light pink)) leads to a prolonged time course of receptor exchange (green, blue, pink lines) compared to unhindered diffusive receptor exchange between PSD and reservoir. **(B, C)** Paired-pulse ratio for a synapse surrounded with a glial sheath at a distance of 40nm containing a transporter coverage at a density of $5{,}000/\mu m^2$. The receptors were stationary (red) or could diffuse while the reservoir had different sizes (green: reservoir inside cleft only, blue: reservoir with extra-cleft part of equal size, pink: the extra-cleft part is twice the size of the intra-cleft part).

5 Effect of reservoir size on pulse trains: Further comments

Figure 5 is the same as Figure 7 in the main article, but shows the effect of a small and a large extra-cleft reservoir of diffusing AMPAR receptors. While the intra-cleft reservoir contains 30 receptors, the small extra-cleft reservoir has twice the size of the intra-cleft reservoir and hence 30 additional receptors, and the large one has fourfold size corresponding to 90 additional receptors.

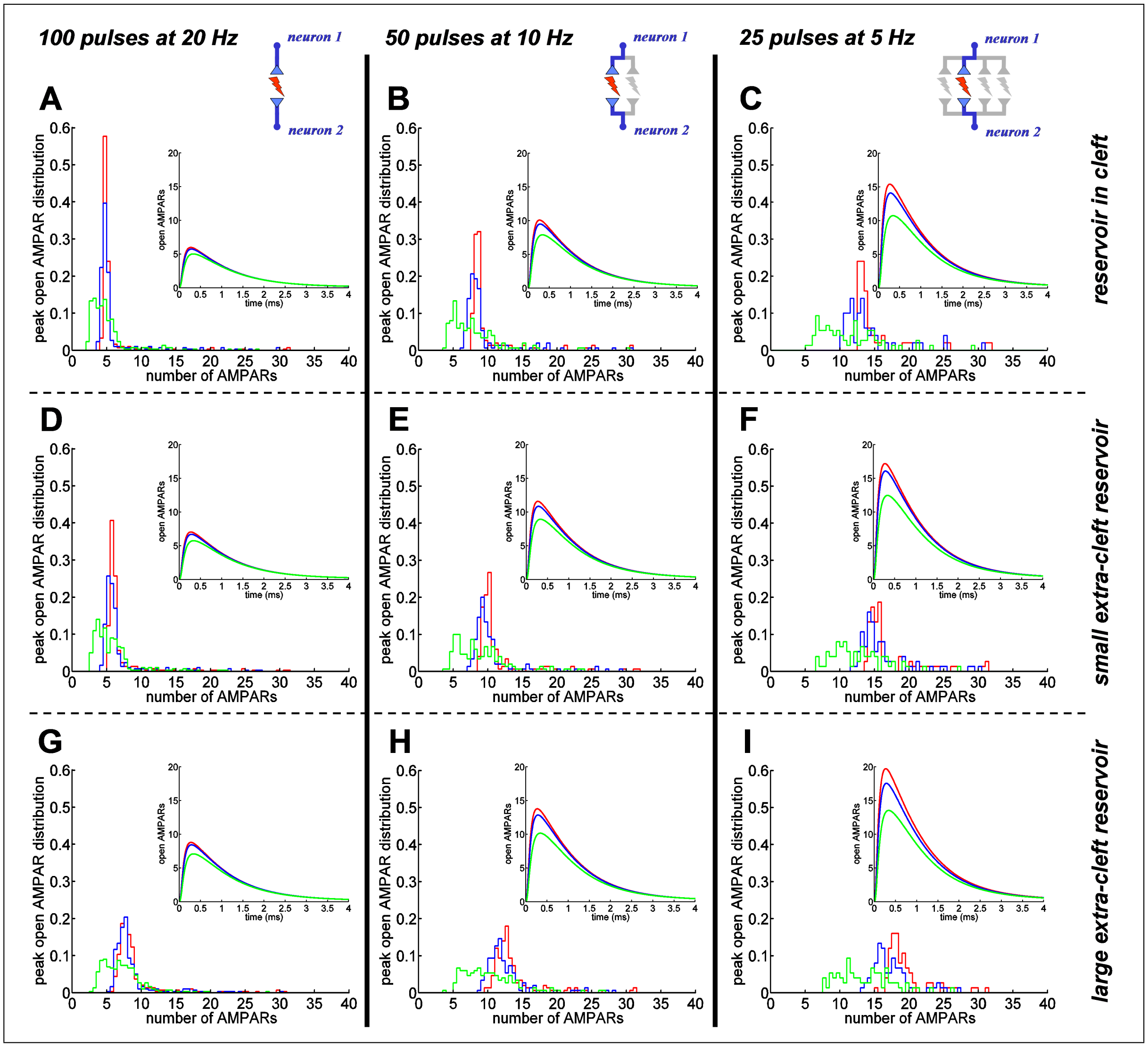

Figure 5: Recovery from postsynaptic depression by spike decorrelation and reservoir enlargement. A spike train at a single synaptic connection can lead to strong postsynaptic depression. The normalized distributions of the maximal number of open AMPARs (for the MN scheme) per pulse at a single participating synapse are shown for different stimulation intensities. Insets: averaged spike-to-spike time course of AMPAR openings. During a single simulated Poissonian spike train, one vesicle was released per pulse where the release sites were 1) clustered at the AZ center (red), 2) uniformly distributed over PSD (blue), 3) uniformly distributed over the cleft (green). Enlarging the AMPAR reservoir from intra-cleft only (A-C) to an additional extra-cleft one (of same size, (D-F); of fourfold size, (G-I)) increases the averaged synaptic response. (100 AMPARs on PSD, AMPAR density in the reservoir 10 times smaller than on the PSD, diameter of PSD 200nm, of cleft 400 nm, of intra-cleft reservoir 200 nm, transporter density $5000/\mu m^2$, cleft height 20 nm, distance ofglial sheath 40 nm).

6 Comparison of the JS, MN, and RL AMPAR kinetic models

We compare here the three kinetic schemes JS, MN, and RL that we have used (see Section 7.2) to model AMPAR dynamics with the help of the time to recover from a glutamate impulse and the percentage of probability density. First, we compute the *deactivation time* $\tau_{deactivation}$, which accounts for the mean time an AMPAR closes, after a square glutamate pulse of 1 mM concentration and lasting 1 ms. The time $\tau_{deactivation}$ is computed as the first time the probability of the open state reaches 10% of its peak value. From the numerical simulations of the kinetic equations (14) (Section 7.2 below) we find (see Fig 6A)

$$\tau_{deactivation} = 7.1\text{ms} \qquad \text{(JS scheme)} \tag{4}$$
$$\tau_{deactivation} = 15.1\text{ms} \qquad \text{(RL scheme)} \tag{5}$$
$$\tau_{deactivation} = 3.0\text{ms} \qquad \text{(MN scheme)} \tag{6}$$

Next, we compute the time *desensitization time* $\tau_{desensitization}$, which is the mean time AMPAR desensitizes after it is exposed to a permanent glutamate concentration of 1 mM. It is the first time the opening probability reaches 10% of its peak amplitude, after the glutamate concentration was raised from 0 to 1 mM at the initial time 0. We find (see Fig 6B)

$$\tau_{desensitization} = 30.3\text{ms} \qquad \text{(JS scheme)} \tag{7}$$
$$\tau_{desensitization} = 45.8\text{ms} \qquad \text{(RL scheme)} \tag{8}$$
$$\tau_{desensitization} = 8.7\text{ms} \qquad \text{(MN scheme)} \tag{9}$$

Finally, we expose a single receptor to a square glutamate pulse of 1 ms duration and 1 mM concentration, and compute the *return probability* p_{return}, which measures the probability of an AMPAR, after 100 ms, not to be in the unbound resting state, to which it will eventually return. We find (see Fig 6C)

$$p_{return} = 0.08 \qquad \text{(JS scheme)} \tag{10}$$
$$p_{return} = 0.33 \qquad \text{(RL scheme)} \tag{11}$$
$$p_{return} = 0.45 \qquad \text{(MN scheme)} \tag{12}$$

These numerical estimates highlight the difference between these AMPAR models.

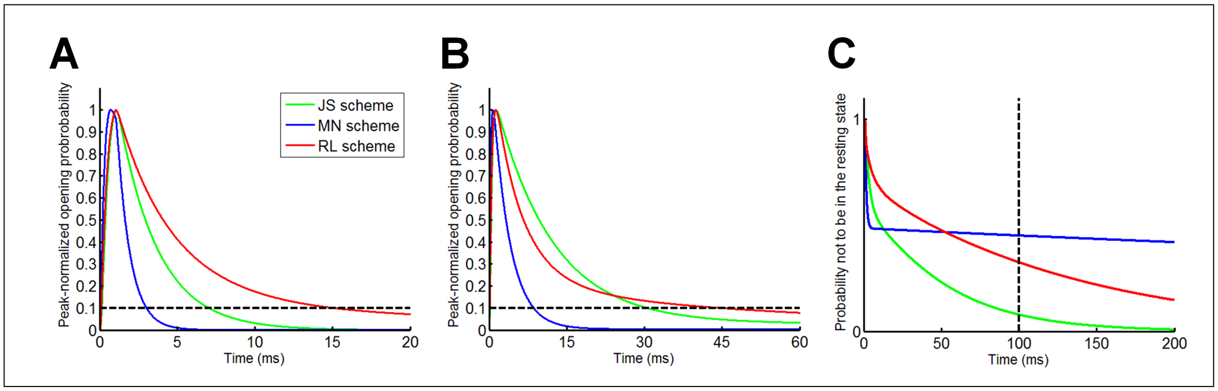

Figure 6: Comparison of the three AMPAR schemes. **(A)** Dynamics of the open state: The time $\tau_{deactivation}$ is computed as the return time to 10% (black line) of the maximal amplitude following a 1 ms glutamate pulse. We find $\tau_{deactivation}$ of 7.1 ms (JS, green), 15.1 ms (RL, red), 3 ms (MN, blue). **(B)** Dynamics of the open state: the desensitization time $\tau_{desensitization}$ is computed from a permanent glutamate concentration signal as the return time to 10% (black line) of the maximal amplitude. It is 30.3 ms (JS, green), 45.8 ms (RL, red), 8.7 ms (MN, blue). **(C)** Dynamics of the return probability p_{return} to the open state of an AMPAR. We sample this probability 100 ms (black line) after a 1 ms glutamate pulse. It is 0.08 (JS, green), 0.33 (RL, red), 0.45 (MN, blue). All curves are normalized to the peak amplitude.

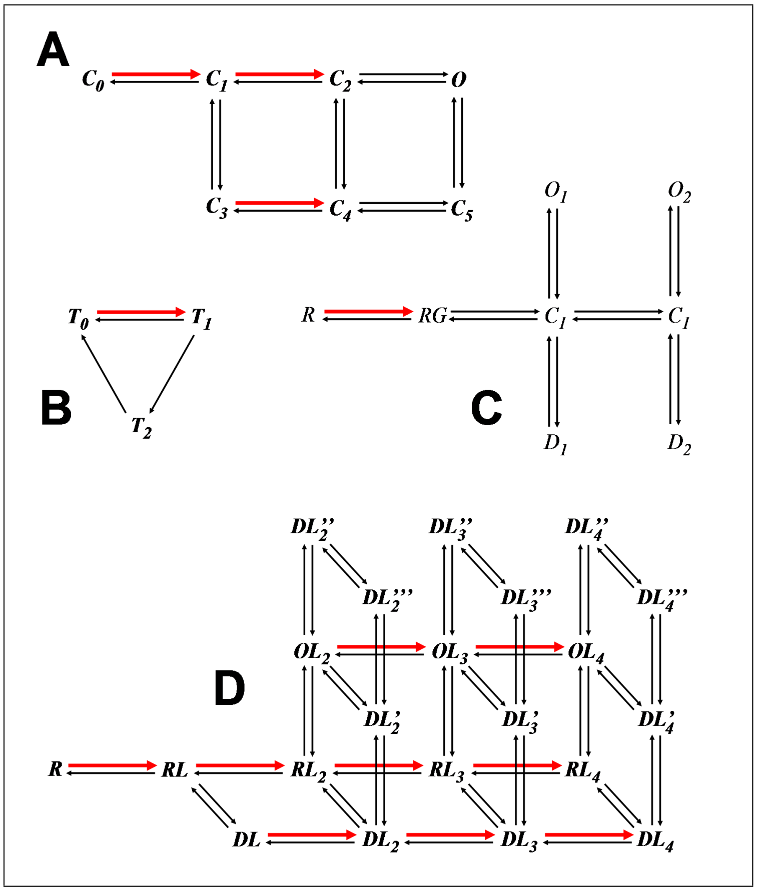

Figure 7: Kinetic schemes for AMPARs and glial transporters. The red arrows refer to transition rates to be multiplied with the glutamate concentration applied. **(A)** The Jonas-Sakmann AMPAR scheme [4]. **(B)** The transporter scheme from [7]. **(C)** The Milstein-Nicoll AMPAR scheme [5] (no TARP ligation). **(D)** The Raghavachari-Lisman AMPAR scheme [6].

The resting state of the AMPA-receptor is denoted by R in all schemes A, C, D.

Rates for the schemes:

JS scheme		MN scheme		Transporter	scheme
$k_{C_0C_1}$	$4.59\,10^6 M^{-1}s^{-1}$	$k_{R,RG}$	$1\,10^7 M^{-1}s^{-1}$	$k_{T_0T_1}$	$1.8\,10^7 M^{-1}s^{-1}$
$k_{C_1C_0}$	$4.26\,10^3 s^{-1}$	$k_{RG,R}$	$5\,10^4 s^{-1}$	$k_{T_1T_0}$	$1.8\,10^9 s^{-1}$
$k_{C_1C_2}$	$2.84\,10^7 M^{-1}s^{-1}$	k_{RG,C_1}	$3.65\,10^4 s^{-1}$	$k_{T_1T_2}$	$1.8\,10^9 s^{-1}$
$k_{C_2C_1}$	$3.26\,10^3 s^{-1}$	$k_{C_1,RG}$	$4.55\,10^3 s^{-1}$	$k_{T_2T_0}$	$2.57\,10^8 s^{-1}$
k_{C_2O}	$4.24\,10^3 s^{-1}$	$k_{C_1C_2}$	$3\,10^2 s^{-1}$		
k_{OC_2}	$9.00\,10^2 s^{-1}$	$k_{C_2C_1}$	$1\,10^4 s^{-1}$		
$k_{C_1C_3}$	$2.89\,10^3 s^{-1}$	$k_{C_1O_1}$	$1\,10^4 s^{-1}$		
$k_{C_3C_1}$	$3.92\,10^1 s^{-1}$	$k_{O_1C_1}$	$6\,10^3 s^{-1}$		
$k_{C_2C_4}$	$1.72\,10^2 s^{-1}$	$k_{C_2O_2}$	$1\,10^4 s^{-1}$		
$k_{C_4C_2}$	$0.727\,s^{-1}$	$k_{O_2C_2}$	$6\,10^3 s^{-1}$		
k_{OC_5}	$1.77\,10^1 s^{-1}$	$k_{C_1D_1}$	$1.1\,10^3 s^{-1}$		
k_{C_5O}	$4.00\,s^{-1}$	$k_{D_1C_1}$	$1\,s^{-1}$		
$k_{C_3C_4}$	$1.27\,10^6 M^{-1}s^{-1}$	$k_{C_2D_2}$	$3\,10^2 s^{-1}$		
$k_{C_4C_3}$	$4.57\,10^1 s^{-1}$	$k_{D_2C_2}$	$10\,s^{-1}$		
$k_{C_4C_5}$	$1.68\,10^1 s^{-1}$				
$k_{C_5C_4}$	$190.4\,s^{-1}$				

7 Simulation analysis and description of algorithms

We describe here the algorithms we used for the simulations of glutamate molecules, AMPAR trafficking and dynamics and the glial transporters.

7.1 Simulation of glutamate diffusion

When the glutamate molecules are freed from a vesicle, they are placed on the release site, represented by a single spot located on the presynaptic terminal. Later on, all glutamate molecules are freely diffusing and we simulate the glutamate trajectories at a time step of $\Delta t = 0.5\mu$s using a first order Euler scheme

$$X_{n+1} = X_n + \sqrt{2D_{glu}\Delta t}\, G_n \tag{13}$$

where G_n are uncorrelated standard Gaussian numbers. Upon hitting a reflecting wall (the pre- or post-synaptic cylinder or glial sheath), trajectories are reflected when hitting point is located on the part of a cell containing the membrane only. However, when a glutamate molecule hits an AMPAR, it is not hold there to describe the binding event, but rather reflected. The behavior of an AMPAR is completely controlled by the number of hits (instead of bindings) per time step. We give more details in Section 7.2.1. Similarly, a glutamate molecule hitting a transporter is either bound, reflected, or pumped into a glial cell, corresponding to the dynamics of the transporter as described in Section 7.2.2.

7.2 Simulation of AMPA-receptors and glial transporters

The dynamics of AMPARs and glutamate transporters were modelled by classical Markov chains. The glutamate dependence of the dynamics is taken into account in the transition rates which are proportional to the concentration in the neighborhood of the receptor. To

every AMPAR or transporter, we use the probability vectors $p_i(t) := \Pr(\text{state } i \text{ at time } t)$ for $i = 1, \ldots, N$ where N is the total number of internal states. The transition probability function $\mathbf{p}(t) = (p_1(t), \ldots, p_N(t))$ satisfies the Markov transition equation

$$\mathbf{p}'(t) = \mathbf{p}(t)\mathbf{Q}\big(C(t)\big) \tag{14}$$

$$\mathbf{p}(0) = \mathbf{p}_{initial} \tag{15}$$

where $\mathbf{p}_{initial}$ is the initial probability conditions and $\mathbf{Q}$ is the transition matrix which depends on the glutamate concentration $C(t)$. Because some transition rates depend on the glutamate concentration, we further characterize the local concentration (amount glutamate near the glutamate binding domain) in the following.

7.2.1 Simulation of AMPAR activation

To simulate AMPAR activation, we have used three different kinetic schemes: The JS model ([4], Fig. 7A), the MN model ([5], Fig. 7C), and the RL model ([6], Fig. 7D)). The JS model consists of 7 Markov states, and was fitted to measurements of hippocampal CA3 pyramidal cells. The MN model has 8 states using the stargazer cerebellar granule neurons (AMPARs without TARP ligation). The RL model has 21 states using CA1 neurons.

In our model, we do not consider that a glutamate molecule can physically interact with a specific binding site; rather, AMPAR activation is triggered by the local concentration $C_l(t)$ of glutamate molecules at an AMPAR. Binding to AMPARs is indeed negligible due to the hight amount of glutamate molecules compared to AMPARs. In practice, for each AMPAR, we compute $\mathbf{p}(t)$ by solving equation (14) with the transition matrix $Q(C_l(t))$ by a Runge-Kutta method.

7.2.2 Simulation of glial transporters

We use a standard kinetic scheme [7] with 3 states (Fig. 7B) to model transporters. If a transporter is in the 'ready-to-bind' state (denoted by T_0 in Fig. 7B), a glutamate molecule can bind to it, causing a reversible transition to a bound state, denoted T_1. Upon reverting, the molecule dissociates and can diffuse away. Otherwise, the transporter transits to a 'pumping-in' state T_2, followed by a transition to the 'ready-to-bind' state, in a time necessary to internalize the molecule into the glial cell. We detail in Section 7.2.4 the calibration of the different parameters. Unless the transporter is bound (i.e. not in the 'ready-to-bind' state) a hit by a glutamate molecule leads to transition to the 'bound' state and the glutamate trajectory is suspended. Otherwise the trajectory is reflected. We compute the transporter state by choosing randomly the waiting time T and, by using a Gillespie algorithm, the next state transition. Upon transition from the 'bound' to the 'pumping-in' state, glutamate trajectories are terminated. Upon reverting to the 'ready-to-bind' state, a glutamate trajectory is continued at a position inside the extra-synaptic space by adding a displacement away from the location of the transporter. The displacement is sampled from a Gaussian with mean 0 and variance $0 \le \tau < \Delta t$, where τ is the time needed to adjust the waiting time T to the next time step (a similar method is described in [1]).

7.2.3 Simulation of AMPAR trafficking and maintaining prescribed AMPAR concentrations in PSD and reservoir

To maintain a fixed number N_{PSD} and N_{res} of AMPARs uniformly distributed on the PSD and in the reservoir, respectively, we used the following numerical protocol: the PSD (resp. reservoir) of size A_{PSD} (resp. A_{res}), have a densities of $\rho_{PSD} = N_{PSD}/A_{PSD}$ (resp. $\rho_{res} = N_{res}/A_{res}$), thus a Brownian receptor crossing from the PSD to the reservoir is accepted with a probability $p = \rho_{res}/(\rho_{PSD} + \rho_{res})$.

7.2.4 The local glutamate concentration

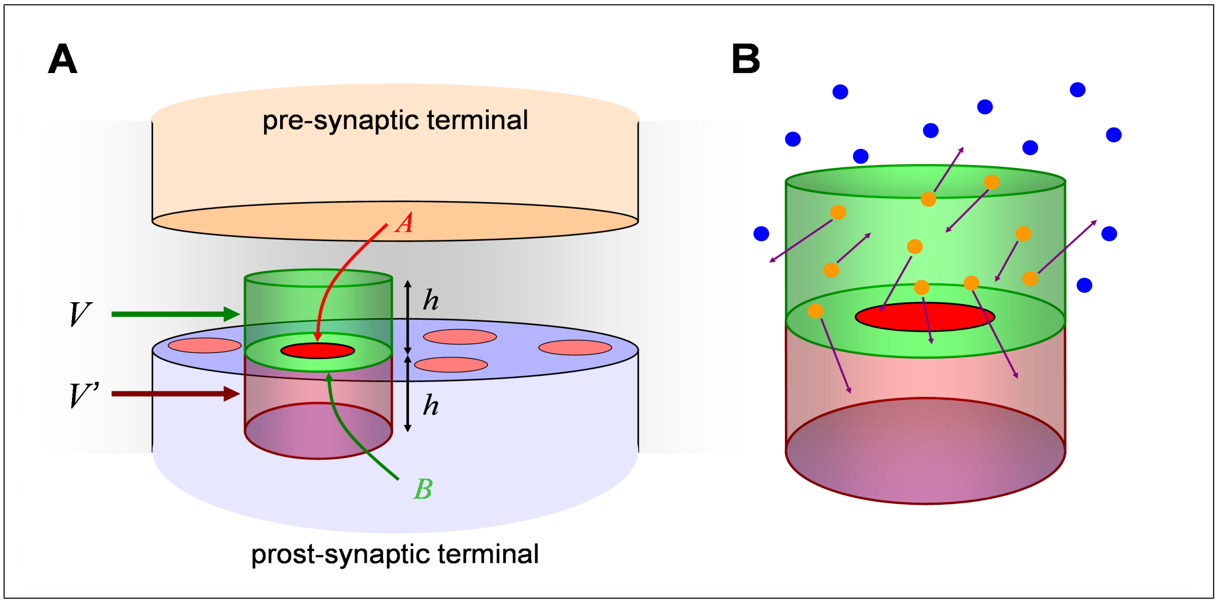

Figure 8: Schematic representation of a glutamate molecule near an AMPA-receptor. (A) Scheme of the synaptic cleft contained a pre and postsynaptic terminal with AMPARs, modeled as round patches. The binding domain of an AMPAR is modelled by a disk A (red, other AMPARs: light red). We choose a cylindrical domain of volume V with a down face B and height h large enough (equal to the mean square displacement) so that it contains some glutamate molecules that can hit the surface A during the next time step. The volume V' is equal to V, but located the associated domain is located below the cleft bottom membrane. **(B)** Released glutamate molecules outside (blue) and inside (orange) the volume V. By reasoning on all molecules inside V that cross from V into V' , we obtain an approximation for the probability of hitting the area A.

We now explain how we computed the probability of AMPARs activation. This computation depends on the local concentration of glutamate molecules in a pillbox of height h, surface B and volume $V = hB$, above a small surface A enclosing a glutamate binding site, (see Fig. 8). We provide further explanations below on how this surface was chosen. To compute the number of hits that a molecule inside the pillbox produces with the surface A, we estimate the probability p_H that a molecule at position X_t in that box hits the surface A during a time interval Δt,

$$p_H = Pr(X_{t+\Delta t} \in V' \text{ and the trajectory hits } A | X_t \in V) \quad (16)$$

where V' is the mirror image of V on the bottom face B. For a molecule found with (approximately) uniform distribution in V,

$$p_H = Pr(X_{t+\Delta t} \in V' \text{ and the trajectory hits } A \,|\, X_t \in V) \tag{17}$$

$$= \frac{A}{B}\int_{-\infty}^{0}\frac{1}{h}\int_{0}^{h} p(2D\Delta t, x-y)dxdy \approx \frac{A}{V}\int_{-\infty}^{0}\int_{0}^{\infty} p(2D\Delta t, x-y)dxdy = \frac{A}{V}\sqrt{\frac{D\Delta t}{\pi}}, \tag{18}$$

(see Fig. 8B.) where $p(\sigma^2, \mu)$ is the Gaussian density with mean μ and variance σ^2. Our approximation holds for Δt small enough so that the mean-square displacement is much less than h (corresponding to $\Delta t < h^2/6D$). When there are N independent molecules, the number H_t of hits on A during Δt follows a binomial distribution with mean and variance

$$\langle H_t \rangle = Np_H = \frac{NPr(X_t \in V)}{V} A\sqrt{\frac{D\Delta t}{\pi}} \tag{19}$$

$$\langle\langle H_t \rangle\rangle = Np_H(1 - P_H). \tag{20}$$

Because $NPr(X_t \in V)/V$ is the probability for glutamate molecules to be in V, we introduce the local concentration for given time step size Δt by

$$C_t = \frac{H_t}{A}\sqrt{\frac{\pi}{D\Delta t}}. \tag{21}$$

such that it has the moments

$$\langle C_t \rangle = \frac{NPr(X_t \in V)}{V} \tag{22}$$

$$\langle\langle C_t \rangle\rangle = \langle C_t \rangle \left(\frac{1}{A}\sqrt{\frac{\pi}{D\Delta t}} - \frac{\langle C_t \rangle}{N} \right). \tag{23}$$

All glutamate molecules are approximately uniformly distributed inside the volume V, (where the length/radius is larger than the mean square displacement of a single glutamate molecule as simulated by Brownian dynamics with fixed time step size Δt). Solving the diffusion equation in the cleft for small times shows that equilibration is reached after three time steps of size $\Delta t = 5 \times 10^{-4}$ ms.

AMPAR activation. The activation of an AMPAR (see Section 7.2.1) depends on the number of molecules in the box V. (Note that C_t as derived above is a volume concentration, and has to be converted to molar concentration for use with the Markov scheme.) Following relation (22), the mean discrete concentration of glutamate molecules is independent of A and converges, as Δt tends to 0, to the continuous concentration. However, from equation (23), it can be seen that the concentration variance depends on the surface A and tends to infinity as Δt becomes small. Therefore, we conducted all simulations with the same A and the same Δt. (Otherwise, a change of Δt has to be accompanied by scaling of A.)

Choosing the surface A for glial transporters. To determine the size of the surface A associated with glutamate binding to a transporter, we use the forward binding rate constant k_{on} for the glutamate molecules. The number of glutamate molecules hitting A during δt is

$$\langle H_t \rangle = k_{on} \langle C_t \rangle \Delta t \,. \tag{24}$$

which we evaluated using expression (19),

$$\langle H_t \rangle = \langle C_t \rangle A \sqrt{D \Delta t} / \sqrt{\pi}, \tag{25}$$

thus, we find that

$$A = k_{on} \sqrt{\frac{\pi \Delta t}{D}} \,. \tag{26}$$

A similar formula was obtained in [2, Formulas (6a), (6b)], [3]. It may happen, however, that A can be too small compared to the mean square displacement of the discretization $\sqrt{2D\Delta t}$ of a single glutamate trajectory. In that case, A has to be enlarged to a disk $\tilde{A}$ with a radius larger than $\sqrt{2D\Delta t}$ and we have to use the rejection probability $A/\tilde{A}$ to single out the additional hits on $\tilde{A}$.

7.2.5 Comparing the Brownian dynamics simulation with a differential equation solver

To test the robustness of our approach, we compare our simulation output obtained from a Monte-Carlo simulation with the ones given by by solving partial differential equations. We fixed the synaptic geometry, in which the glial sheath was far away from the synapse such that we could consider that molecules are lost once they had left the cleft. We placed a single release site at the center of the active zone. The cleft and PSD diameter are respectively 440 nm and 400 nm. We use 400 AMPARs located on the PSD, none in the reservoir, and a single vesicle releases 3000 molecules.

We use an average local concentration C at an AMPAR located at the point x, C is the sum of all realization averages of trajectories of N glutamate molecules in x and connected to the probability $p(t, x)$ of a single glutamate molecule to be in $x + dx$ at time t as

$$C(t, x) = N p(t, x) \,. \tag{27}$$

Then $C(t, x)$ satisfies the diffusion equation

$$\partial_t C(t, x) = D \Delta_x C(t, x) \tag{28}$$
$$C(0, x) = N \delta(x - 0), \tag{29}$$

with an absorbing boundary condition on the cleft boundary $\{|x| = R_{cleft}\}$. Due to fast equilibration, we can average in the cleft height coordinate, and we hence consider the

diffusion in the two dimensional disk $\{|x| < R_{cleft}\}$. The solution $C(t,r)$ (with r being the radial distance to the cleft middle axis) is given by

$$C(t,r) = N\left(-\frac{2}{h\pi R_{cleft}^2}\sum_{n=0}^{\infty}\frac{J_0\big(j_n\frac{r}{R_{cleft}}\big)}{J_1(j_n)^2}J_0\big(j_n\frac{r}{R_{cleft}}\big)e^{-D\frac{j_n^2}{R_{cleft}^2}t}\right) \tag{30}$$

where J_0, J_1 are the Bessel functions of the first kind and order 0 and 1, respectively, and j_n is the ascending sequence of zeros of J_1. Now it is possible to solve the AMPAR kinetic equations (14) using the concentration $C(t,r)$, where r is the radial distance of the AMPAR to the PSD center. We solved (14) with a Runge-Kutta method for both the JS and MN schemes and compare in Fig. 9our results to the Brownian dynamics method. We obtained good agreement.

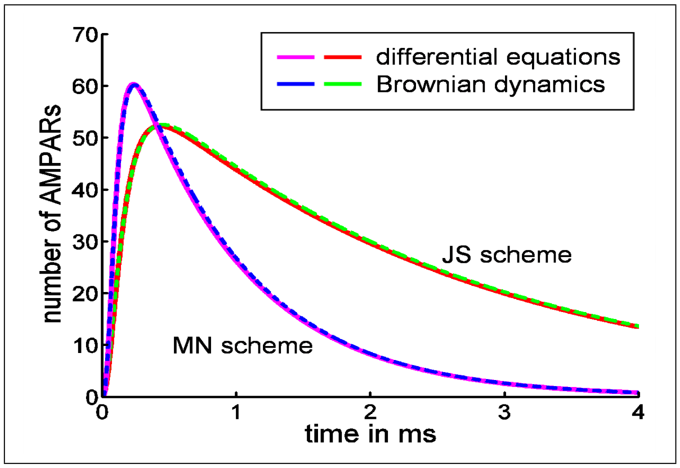

Figure 9: Comparison of the Brownian simulations with the analytical solution. Simulation of the mean open AMPAR number versus time in our Brownian dynamics (BD) coupled to the Runge-Kutta-Fehlberg method (RKF). The differences for the two AMPAR models used in this study, the JS scheme (red: RKF, green: BD) and the MN scheme (pink: RKF, blue: BD), are negligibly small (less then 1 AMPAR). Synapse parameters: Cleft diameter 440 nm, PSD diameter 400 nm, 400 AMPARs on the PSD, none in the reservoir, no receptor diffusion, glutamate leaving the cleft is considered lost.

References

[1] Singer A, Schuss Z. Brownian simulations and unidirectional flux in diffusion. Phys.Rev.E 71, 026115 (2005)

[2] Bartol TM, Land BR, Salpeter EE, Salpeter MM. Monte Carlo simulation of miniature endplate current generation in the vertebrate neuromuscular junction. Biophys.J. 1991;59:1290-1307

[3] A Singer, Z Schuss, A Osipov, D Holcman, *Partially Reflected Diffusion,* SIAM J.Appl.Math. 68, 844-868 (2008)

[4] Jonas P, Major G, Sakmann B. Quantal components of unitary EPSCs at the mossy fibre synapse on CA3 pyramidal cells of rat hippocampus. J.Physiol. 1993;472:615-663

[5] Milstein AD, Zhou W, Karimzadegan S, Bredt DS, Nicoll RA. TARP subtypes differentially and dose-dependently control synaptic AMPA receptor gating. Neuron 2007;55(6):905-18.

[6] Raghavachari S, Lisman JE. Properties of quantal transmission at CA1 synapses. J Neurophysiol. 2004;92(4):2456-67.

[7] RM Franks, CF Stevens, TJ Sejnowski. Independent sources of quantal variability at single glutameric synapses. J. Neur. 23(8) pp.3186-3195 (2003)

[8] K Zheng, A Scimemi, DA Rusakov. Receptor Actions of Synaptically Released Glutamate: The Role of Transporters on the Scale from Nanometers to Microns. Biophys J. 95(10) pp.4584-96 (2008)
